# Multiple Integrated Non-clinical Studies Predict the Safety of Lentivirus-Mediated Gene Therapy for β-Thalassemia

**DOI:** 10.1016/j.omtm.2018.09.001

**Published:** 2018-09-13

**Authors:** Maria Rosa Lidonnici, Ylenia Paleari, Francesca Tiboni, Giacomo Mandelli, Claudia Rossi, Michela Vezzoli, Annamaria Aprile, Carsten Werner Lederer, Alessandro Ambrosi, Franck Chanut, Francesca Sanvito, Andrea Calabria, Valentina Poletti, Fulvio Mavilio, Eugenio Montini, Luigi Naldini, Patrizia Cristofori, Giuliana Ferrari

**Affiliations:** 1 San Raffaele Telethon Institute for Gene Therapy (SR-TIGET), IRCCS San Raffaele Scientific Institute, Milan, Italy; 2 Department of Molecular Genetics Thalassaemia, The Cyprus Institute of Neurology and Genetics, Nicosia, Cyprus; 3 Vita Salute San Raffaele University, Milan, Italy; 4 GlaxoSmithKline Ware, Hertfordshire, UK; 5 Department of Pathology, IRCCS San Raffaele Scientific Institute, Milan, Italy; 6 Genethon, Evry, France; 7 Department of Life Sciences, University of Modena and Reggio Emilia, Modeno, Italy

**Keywords:** thalassemia, gene therapy, hematopoiesis, hematopoietic stem cell, preclinical model, safety, lentiviral vector, biodistribution, genotoxicity, integration site

## Abstract

Gene therapy clinical trials require rigorous non-clinical studies in the most relevant models to assess the benefit-to-risk ratio. To support the clinical development of gene therapy for β-thalassemia, we performed *in vitro* and *in vivo* studies for prediction of safety. First we developed newly GLOBE-derived vectors that were tested for their transcriptional activity and potential interference with the expression of surrounding genes. Because these vectors did not show significant advantages, GLOBE lentiviral vector (LV) was elected for further safety characterization. To support the use of hematopoietic stem cells (HSCs) transduced by GLOBE LV for the treatment of β-thalassemia, we conducted toxicology, tumorigenicity, and biodistribution studies in compliance with the OECD Principles of Good Laboratory Practice. We demonstrated a lack of toxicity and tumorigenic potential associated with GLOBE LV-transduced cells. Vector integration site (IS) studies demonstrated that both murine and human transduced HSCs retain self-renewal capacity and generate new blood cell progeny in the absence of clonal dominance. Moreover, IS analysis showed an absence of enrichment in cancer-related genes, and the genes targeted by GLOBE LV in human HSCs are well known sites of integration, as seen in other lentiviral gene therapy trials, and have not been associated with clonal expansion. Taken together, these integrated studies provide safety data supporting the clinical application of GLOBE-mediated gene therapy for β-thalassemia.

## Introduction

The development of retroviral vectors and optimized transduction protocols has permitted gene transfer into hematopoietic stem and progenitor cells (HSPCs), leading to the application and translation of gene therapy strategies for a number of hematopoietic diseases and leukodystrophies (reviewed in Dunbar et al.,^1^ Eichler et al.,^2^ Aiuti et al.,^3^ Biffi et al.,^4^ and Ribeil et al.^5^).

β-Thalassemia, an inherited anemia characterized by reduced or absent production of hemoglobin β chains, was one of the first candidate diseases for gene therapy.^6^ Current management strategies for transfusion-dependent thalassemia patients include blood transfusion, iron chelation, and splenectomy. To date, allogeneic hematopoietic stem cell transplantation (HSCT) is a cure only available for less than 30% of patients.^7^ The benefits and limitations of current therapies for β-thalassemia are well discussed by Cappellini et al.^8^ *Ex vivo* engineering of autologous HSPCs and administration of genetically modified cells potentially represents a cure applicable to all patients regardless of donor availability and free from transplant-related immunological complications such as graft rejection and graft versus host disease. The evidence gained from allogeneic HSCT indicates that 20%–30% of donor chimerism can be curative in β-thalassemia patients and provides the rationale for a gene therapy approach.^9^ A recent study of 4 patients treated with HSCT demonstrated that, despite low myeloid chimerism, the majority of circulating erythrocytes and progenitors were of donor origin, suggesting that they have a selective advantage in *vivo*.^10^ These data indicate that the proportion of genetically modified nucleated progenitor cells necessary to achieve a therapeutic level of circulating red blood cells (RBCs) is within the frequency of gene transfer by β-globin lentiviral vector (LV).^10^ Furthermore, non-clinical studies conducted in human HSPCs *in vitro* and murine models *in vivo*, as well as the first clinical trial, have demonstrated the feasibility of the gene therapy approach for the treatment of β-thalassemia.^11^, ^12^

Despite the therapeutic success in clinical trials, lymphoproliferative disorders and pre-malignant expansion of myeloid progenitors have been reported in some patients treated with HSPCs transduced with Moloney murine leukemia virus (MoMLV)-derived vectors, where the retroviral vector was inserted into and activated a proto-oncogene.^13^, ^14^, ^15^ Proviral elements that are involved in the transcriptional regulation of the provirus can alter gene transcription; in particular, elements present in the long terminal repeat (LTR) and the splice donor sites normally used for splicing of viral transcripts. Certain genotoxic events could lead to activation of the transforming potential of a cellular proto-oncogene after integration of a retroviral vector within or near the proto-oncogene, as reviewed by Naldini.^16^ Non-clinical studies showed that LVs are less likely to cause insertional gene activation than retroviral vectors,^17^ although they can still cause interference with normal gene regulation by inducing alterations in transcriptional activity,^18^, ^19^, ^20^, ^21^ aberrant splicing,^22^, ^23^ and premature transcript termination, as observed in the first clinical trial of gene therapy for β-thalassemia.^11^ In this study, a clone harboring an insertion site located within the proto-oncogene HMGA2 accounted for 30% of all integration sites (ISs) in whole blood cells 3 years after gene therapy. In the erythroid cells, the integration event was associated with the expression of an HMGA2 transcript whose 3′ UTR was replaced by vector sequences. This chimeric transcript lacked the target site of the repressive miRNA let-7, contributing to elevated HMGA2 expression together with an increase in the rate of transcriptional initiation. Although the treated patient still remains healthy 11 years after therapy, HMGA2 has been found to be de-regulated in some cancers.

Indeed, understanding the interactions between viral vectors and the genome is instructive to assess the characteristics of transduced cells, although the contribution of different clones to hematopoietic reconstitution is not predictable from non-clinical studies. In gene therapy for β-thalassemia, genotoxicity by *trans*-activation is expected to be very limited because of the erythroid-restricted activity of promoter and/or enhancer elements included in the self-inactivating vector and because of the nature of the transferred gene (human β-globin chain). No report of general toxicity or development of tumors associated with the transfer of the human β-globin gene by LV has been reported so far in a large number of non-clinical studies in mouse models.^24^, ^25^, ^26^, ^27^, ^28^, ^29^ Therefore, in view of future clinical application, safety studies of genetically modified cells are preferably conducted following guidelines issued by the regulatory agencies such as the European Medicines Agency (EMA) and the US Food and Drug Administration (FDA). Crucial parameters to be assessed, when appropriate and feasible, include the toxicity of the vector, the profile of vector integration, the *in vivo* biodistribution of transduced cells, and germline transmission. Although *in vitro* tests for transduced HSPCs are defined, surrogate *in vivo* assays may rely on transplantation of transduced cells in murine strains and/or humanized immunodeficient mouse models.^30^, ^31^, ^32^, ^33^, ^34^, ^35^

We have previously reported the development of GLOBE LV and demonstrated expression of therapeutic levels of β-globin and long-term correction with *in vivo* selection of genetically corrected erythroid cells in a severe β-thalassemia intermedia mouse model^26^ as well as restoration of normal erythroid differentiation from transduced CD34^+^ cells of β-thalassemia patients.^36^ In both mouse and human cells, we obtained proof of efficacy with the achievement of therapeutic levels of β-globin expression with a low vector copy number and in the presence of a limited proportion of transduced HSPCs.

To move forward with clinical development, we identified the best-performing β-globin LV by comparing GLOBE and newly derived vectors for their transcriptional activity and potential interference with the expression of surrounding genes. Furthermore, to support the use of HSCs transduced by GLOBE LV for the treatment of β-thalassemia, we followed the Guideline on the Non-clinical Studies Required before First Clinical Use of Gene Therapy Medicinal Products (EMEA/CHMP/GTWP/125459/2006), which defines scientific principles and provides guidance to applicants developing gene therapy medicinal products to facilitate a harmonized approach in the European Union (EU). To guarantee quality, robustness, and traceability, we designed and performed toxicology, tumorigenicity, and biodistribution studies following the Guidelines for GLP (Good Laboratory Practice) in compliance with the Italian GLP Regulations (DL 50, March 2, 2007; G.U. 8, April 13, 2007) and the Organisation for Economic Co-operation and Development (OECD) Principles of GLP (as revised in 1997, ENV/MC/CHEM(98)17). We evaluated the toxic and oncogenic potential associated with the administration of HSPCs transduced with a high dose of GLOBE LV to C57BL6/Hbb^th3^ mutant (*th3/+*) mice, which represents the homologous disease model of severe thalassemia intermedia. Moreover, to assess the effect of vector transduction on the ability of human CD34^+^ cells to engraft and differentiate correctly in the hematopoietic districts, we transplanted GLOBE LV-transduced CD34^+^ cells into an immunodeficient mouse strain (non-obese diabetic [NOD]/ NOD.Cg-Prkdcsid Il2rgtm1Wjl/SzJ [NSG]) and followed their distribution and differentiation in hematopoietic and non-hematopoietic organs. Both studies were supplemented by vector IS analysis to corroborate safety predictions and HSC-related information.

Overall, our integrated approach, including *in vitro* and *in vivo* experiments, provides results predictive of safety for β-thalassemia gene therapy, complying with the applicable regulatory requirements for advanced medicinal products.

## Results

### Analysis of Perturbation of Expression in Genes Near or Containing GLOBE LV

Changes in the expression of host genes flanking lentiviral ISs are a safety concern when associated with changes in cell biology. To investigate the transcriptional perturbation of genes targeted by GLOBE LV integrations, we utilized human erythroleukemia (HEL) cells, which are permissive but negative for β-globin endogenous expression. HEL cells were transduced with three different LVs, specifically GLOBE, GATA-GLOBE, and cHS4-GLOBE, a derived vector containing the chicken HS4 insulator element in the 3′ LTR, and grown as single clones. Specifically, GATA-GLOBE is a derived vector containing, in the 3′ LTR, the erythroid enhancer GATA1 HS2 element, potentially acting as an insulator, as we reported previously.^37^ The strategy of including insulator elements in the vector LTR aimed to provide protection from the effect of surrounding chromatin to integrated proviruses to obtain reduction of variability and improvement in the β-globin expression level in genetically modified cells. Southern blot analysis of DNA extracted from clones showed that the addition of GATA1-HS2 and cHS4 elements did not affect the stability of the GLOBE LV (data not shown). The vector copy number (VCN) per cell from single clones was measured, and no difference was detected among the three vectors (Figure S1A), indicating a similar transduction ability.

**Figure 1:**
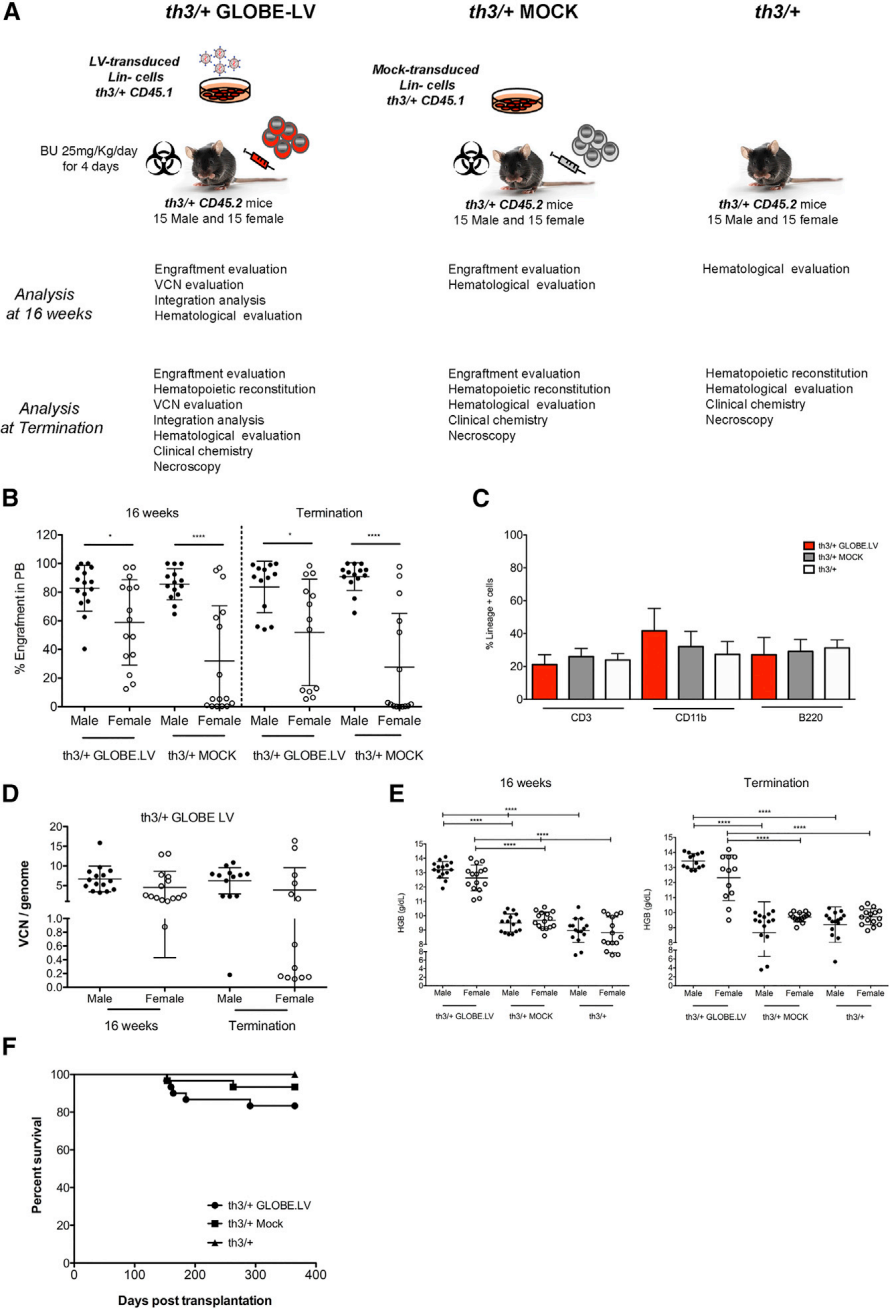
Toxicology and Tumorigenicity Study in a Thalassemia Murine Model (A) Experimental plan of the toxicology and tumorigenicity study. Lin^−^ cells from donor C57BL6/Hbb^th3^ mutant heterozygous (*th3/+*) mice were MOCK-transduced or transduced with GLOBE LV at MOI 100 and incubated overnight. The recipient mice were pre-treated with busulfan (25 mg/kg/day for 4 days). Mice were intravenously injected with a dose of 4 × 10^5^ Lin^−^ cells/mouse. Test group consisted of mice transplanted with GLOBE LV-transduced Lin^−^ cells (*th3/+* GLOBE LV). Control groups consisted of mice pre-treated with busulfan and transplanted with MOCK-transduced Lin^−^ cells (*th3/+* MOCK) and age-matched untreated mice (*th3/+*). Chimerism and cell origin were monitored using antibodies specific for CD45.1 or CD45.2 surface antigen. (B) Donor cell engraftment in male and female recipients was determined by cytofluorimetric analysis for the expression of CD45.1 in peripheral blood mononuclear cells (PBMCs) at 16 weeks and 12 months after transplantation (termination). Values are shown as mean ± SD. Student’s t test was applied (*p < 0.05, ****p < 0.0001). (C) Analysis of hematological reconstitution on peripheral blood was assessed by cytofluorimetric analysis. Percentages of myeloid and lymphoid (B and T) cells were evaluated by using antibodies against CD11b, B220, and CD3, respectively. Values are shown as mean ± SD. The GLOBE LV th3/+ group is shown in red, the MOCK th3/+ group in gray, and the th3/+ group in white. (D) VCN per genome was evaluated in GLOBE LV th3/+ group males and females. Values are shown as mean ± SD. (E) HGB concentration was evaluated in all groups of mice. Values are shown as mean ± SD. One-way ANOVA and *post hoc* Bonferroni’s multiple comparisons were applied (****p < 0.0001). (F) All animals were observed for signs of ill health or overt toxicity from day1 until the end of the study. Mortality was observed in the GLOBE LV th3/+ and MOCK th3/+ groups from day 153 onward. 5 deaths occurred in the GLOBE LV th3/+ group (circles). In the MOCK th3/+ group (squares), two animals died. No mortality was observed in the th3/+ group (triangles). Survival fractions were calculated using the product limit (Kaplan-Meier) method. Survival curves were compared using the log rank test (also called the Mantel-Cox test). No statistical difference was observed between the groups.

The expression of the β-globin transgene was analyzed by qPCR and western blot in HEL cells induced to erythroid differentiation by hemin exposure and normalized for the VCN (Figures 1B and 1C). No difference in transcriptional level was observed between GLOBE and cHS4-GLOBE (Figure S1B), whereas GATA-GLOBE LV produced 4-fold more β-globin transcript than GLOBE LV (p < 0.001). However, the transcriptional difference between GLOBE and GATA-GLOBE LV was not confirmed at the protein level (Figure S1C).

To identify vector ISs, HEL cell clones were subjected to ligation-mediated (LM)-PCR and DNA sequencing. Change in expression level of target genes because of transcriptional activity at viral ISs was analyzed by low-density array (LDA) real-time PCR technology (Applied Biosystems). Genes sited within a window of 200 kb around the IS were included in this analysis. The transcriptional activity of genes was assessed in HEL clones before (uninduced) and after induction to erythroid differentiation (induced) (Table S1). HEL cells are capable of both spontaneous and induced globin expression and, thus, are often used as an erythroid model system.^38^ β-Globin chains are absent, and this pattern of globin synthesis is observed after 4–5 days of induction by hemin. To determine individual deregulated genes in association with viral ISs, the *Z* score test with an adj. p < 0.001 was employed. There were no differences in the frequency of the deregulated target genes among all LVs observed. In uninduced clones, there were 4, 6, and 1 deregulated genes associated with viral integration of GLOBE, GATA-GLOBE, and cHS4 LVs, respectively (Figure S1D; Table S1). In induced clones, there were 6, 1, and 6 deregulated genes associated with viral integration of GLOBE, GATA-GLOBE, and cHS4 LVs, respectively (Figure S1E; Table S1). Considering that there are no significant advantages of the newly derived vectors and given the long development and deep characterization of the GLOBE LV,^22^ we elected to continue the safety characterization with GLOBE LV.

### GLOBE LV-Transduced Cells Correct Thalassemia in the Murine Model

To evaluate the effect of GLOBE LV transduction on hematopoietic cells *in vivo*, we designed and performed a bone marrow (BM) transplantation study in a mouse model of β-thalassemia (th3/+ strain, in compliance with OECD principles and GLP (Figure 1A). The GLOBE LV batch used in this study was produced at medium scale and purified by MolMed with a titer of 9.69 × 10^7^ transduction units (TU)/mL. Lin^−^ cells from th3/+ mice were MOCK-transduced (th3/+ MOCK) or transduced with GLOBE LV (th3/+ GLOBE LV) at an MOI of 100 and transplanted into female and male recipient th3/+ mice (n = 30 per group), which were monitored for up to 12 months after gene therapy. An age-matched untreated control group was included in the study (th3/+, n = 30).

The transduction protocol was designed to achieve a high VCN superior to that expected in human cells (VCN ≥ 2). The transduction efficiency was 100%, and the mean VCN in liquid culture and colony-forming units (CFUs) ranged from 6.03 to 9.69 (number of experiments = 3) (Table S2A).

Recipient mice were pre-treated with a myeloablative dose of busulfan, an alkylating agent causing myeloablation to favor the engraftment of donor cells in recipient HSCs, and transplanted with 0.4 × 10^5^ th3/+ Lin^−^ cells/mouse, equivalent to approximately 25 × 10^6^ human CD34^+^ cells/kg.

Donor and recipient hematopoietic cells were derived from two co-isogenic strains and could be distinguished by alternative expression of the allelic form of the pan-leukocyte surface marker CD45, being CD45.1^+^ and CD45.2^+^, respectively, and detected by cytofluorimetric analysis. Engraftment of th3/+ GLOBE LV− cells and th3/+ MOCK cells was evaluated 16 weeks after transplantation and at termination in peripheral blood (PB). Overall, engraftment percentages (mean ± SD) did not significantly change in th3/+ GLOBE LV mice (70.78 ± 26.47 at 16 weeks) versus th3/+ MOCK mice (56.93 ± 39.56 at 16 weeks) (Tables S2B and S2C) and were maintained over time (67.78 ± 32.85 to 58.14 ± 42.20, respectively) (Figures 1B and 1C; Tables S2B and S2C). The difference in engraftment percentages between female and male, observed in the th3/+ GLOBE LV group (82.71 ± 16.01 male versus 58.87 ± 29.83 female at 16 weeks; 83.64 ± 17.92 male versus 51.93 ± 37.16 female at termination) was not vector-related because it was also present in th3/+ MOCK mice (Figure 1B). The immunophenotype analysis of blood nucleated cells for the percentage of cells expressing B, T lymphoid, and myeloid-specific surface markers (B220, CD3, and CD11b, respectively) showed a normal pattern of lineage development and no evidence of abnormal expansion (Figure 1C). High VCN (mean ± SD) was detected in PB at 16 weeks (6.686 ± 0.8421 male; 4.535 ± 1.060 female) and in BM cells at termination (6.224 ± 0.9259 male; 3.868 ± 1.576 female), demonstrating sustained long-term engraftment of gene-modified cells (Figure 1C; Table S2B). No statistical difference in VCN was observed between the genders. Overall, these data indicate that transduced cells were able to engraft at a high level with no alteration in the hematopoietic reconstitution.

Transplantation of GLOBE-transduced HSPCs in the mouse model of β-thalassemia showed treatment-related efficacy in terms of rescue of anemia, with increased hemoglobin levels, RBCs, hematocrit (HCT), and amelioration of RBC-related parameters (mean corpuscular volume [MCV], mean corpuscular hemoglobin [MCH], and MCH concentration [MCHC]) (Tables S3A–S3C). A low RBC mass level (RBC count, hemoglobin [HGB], and HCT), consistent with clinical anemia present in th3/+ mice, was observed in control groups (th3/+ MOCK and th3/+ mice) (Tables S3B and S3C; Figure 1E). In contrast, th3/+ mice, receiving GLOBE LV gene therapy, showed an increase in RBC mass levels toward normal circulating levels at both sampling times, indicating a treatment-related response (Table S3A; data not shown). The mean RBC count was 6.4–6.8 × 10^6^/mm^3^ in control groups and 8.5–9.3 × 10^6^/mm^3^ in th3/+ GLOBE LV mice and a mean HGB from 8.7–9.7 g/dL to 12.3–13.3 g/dL and mean HCT from 25.0%–28.7% to 35.8%–40.5% (Table S3A; Figure 1E). A correlation between percentages of transduced engrafted cells and transgene expression, measured by hematological analysis (HGB), was observed in most of the cases (Figures 1A, 1D, and 1E). Because VCN analysis is performed on nucleated blood and BM cells, and CD45^+^ expression is absent in the erythroid lineage, in a few cases, a therapeutic level of transgene expression in the presence of a low frequency of transduced cells and/or low VCN was detected as a consequence of positive selection of corrected erythroid cells over the untransduced ones. As in a previous study,^26^ correction of hematological parameters was achieved even in mice with a frequency of BM-transduced cells of less than 15%, presumably because of positive selection of erythroid progenitors carrying productive LV integrations. The differences in all hematological parameters in gene therapy-treated mice versus those in control groups were statistically significant (GLOBE LV versus th3/+ MOCK and GLOBE LV versus th3/+, p < 0.0001), indicating correction of disease-related abnormalities provided by the gene therapy treatment. Increased hemoglobin levels compared with control groups were observed in all mice at both time points (16 weeks and 12 months after treatment), with the exception of animals 952 and 620 at termination, which also showed a low percentage of transduced cells (Figure 1E; Table S3A). Interestingly, in th3/+ MOCK and untreated mice, extramedullary hematopoiesis (EMH) is present in the spleen and liver, with occurrence of related pigmented macrophages or Kupffer cells. In comparison with both aforementioned groups, histological analysis of mice transplanted with GLOBE LV-transduced Lin^−^ HSPCs showed a reduction of these cells (Figure S2).

### Evaluation of Toxicity and Tumorigenic Potential of GLOBE LV-Transduced Murine HSCs

To assess the potential toxicity and/or tumorigenicity of the transplantation of GLOBE LV-transduced cells, mortality and clinical signs were monitored during the 12-month observation period; clinical chemistry, hematologic parameters, necropsy, and full microscopic examination were performed at termination. A board-certified pathologist performed the microscopic examination of a full list of organs of all animals. A peer review of the histopathological analysis was carried out by a second board-certified pathologist.

Differences in survival among the groups were not statistically significant (Figure 1F). No mortality related to the transplantation procedure of Lin^−^ HSPCs transduced with GLOBE LV or MOCK-transduced were observed in the first 4 weeks after treatment. Mortality was observed in the th3/+ GLOBE LV and th3/+ MOCK groups from day 154 onward. There were 7 premature decedents, 5 from the th3/+ GLOBE LV group and 2 from the th3/+ MOCK group (Figure 1F). Deaths were associated with generalized lymphoma in 2 animals in the th3/+ GLOBE LV group and in 1 animal in the th3/+ MOCK group (Tables 1 and 2). Only in one instance was the cause of death unknown.

**Table 1:** Incidence of Tumors Observed in Both Decedents and/or Pre-terminally Sacrificed Animals and at Termination

	Males			Females		
Group (n = 15/sex)	th3/+ GLOBE LV	th3/+ MOCK	th3/+	th3/+ GLOBE LV	th3/+ MOCK	th3/+
Lymphoma (various organs)	1 (ICD)	0	3 (TK)	2 (ICD +TK)	2 (ICD+ TK)	2 (TK)
Histiocytic sarcoma (various organs)	1	0	0	0	0	0
Sex cord stromal tumor in the ovary	0	0	0	1 (TK)	0	0
Bronchiolo-alveolar adenoma in the lungs	0	0	0	0	0	1 (TK)
Sarcoma (not otherwise specified) abdominal	0	1 (ICD)	0	1 (ICD)	0	0

ICD, intercurrent death (days after transplantation); TK, terminal kill sacrificed at 50 ± 1 week.

**Table 2:** Distribution of Hemolymphoproliferative Neoplasia in Both Decedents and/or Pre-terminally Sacrificed Animals and at Termination

Group (n = 15)	Animal Number and Sex	ICD or TK (days)	Lymphomas	VCN PB (BM)	VCN Tissue	% CD45.1 /(CD45.1+Cd45.2) in PB^a^
th3/+ GLOBE LV	617 F	ICD (154)	lymphoma systemic	12.92 (16.53)	8.69	67.3
	690 M	ICD (181)	lymphoma systemic	15.87 (NA)	7.82	80.9
	627 F	TK	lymphoma systemic	6.2 (0.14)	NA	12.8
	605 M	TK	histiocytic sarcoma systemic	7.44 (10.87)	NA	88.1
th3/+ MOCK	654 F	ICD (260)	lymphoma systemic	–	–	62.2
	647 F	TK	lymphoma lung	–	–	0.2
th3/+	661 M	TK	lymphoma mesenteric lymph node	–	–	–
	664 M	TK	lymphoma Peyer’s patch jejunum	–	–	–
	665 M	TK	lymphoma mesenteric lymph node	–	–	–
	677 F	TK	lymphoma Peyer’s patch ileum	–	–	–
	689 F	TK	lymphoma liver	–	–	–

ICD, intercurrent death (days after transplantation); TK, terminal kill sacrificed at 50 ± 1 week; NA, not available; F, female; M, male; –, not applicable.

a Percent chimerism in PB at the latest time point.

Abdominal sarcomas in 1 animal in the th3/+ GLOBE LV group and 1 animal in th3/+ MOCK group as well (Table 1) were considered to be related to the intraperitoneal injection of busulfan in an acetone and peanut oil solution and secondary to the local inflammation induced by the irritation.^39^, ^40^ In another case, abnormal dental growth was the likely cause of decreased food intake, resulting in premature death in the th3/+ GLOBE LV group. No mortality was observed in the untreated th3/+ control group (Figure 1F).

Some tumors were recorded throughout all groups with no difference in tumor incidence or type between the groups, and they were all considered within the normal background incidence in th3/+ mice of this age (Table 1). No signs of toxicity and tumorigenicity related to transplantation of GLOBE LV transduced cells were observed.

Clinical chemistry parameters were evaluated at termination in all groups to detect potential toxic effects in the liver, kidney, pancreas, gut, and cardio-circulatory system. No signs of toxicity were found, and no difference was detected between the three groups in any parameter (Figure S3).

### Hematopoietic Reconstitution in Gene Therapy-Treated Mice Is Highly Polyclonal and Dynamic

To assess the potential genotoxicity of the GLOBE LV and to analyze the clonal dynamics of hematopoietic repopulation in the transplanted mice, we performed a molecular analysis by retrieving the vector genomic integrations in PB- and BM-derived cells 16 weeks and 1 year after transplantation, respectively. We also analyzed the *in vitro* products, both liquid culture and CFUs, 10 days after transduction.

**Figure 2:**
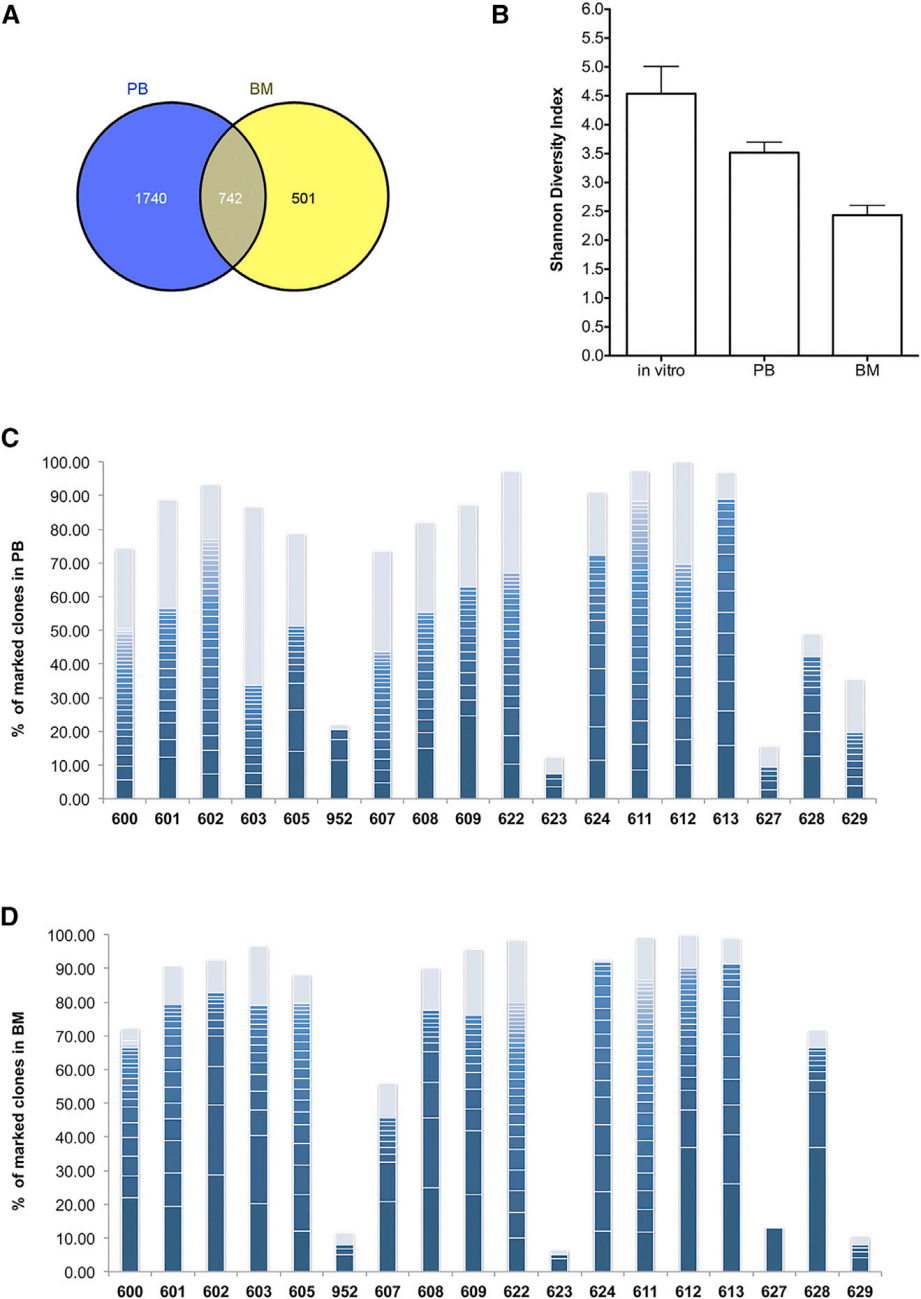
Hematopoietic Reconstitution of Gene Therapy-Treated th3/+ Mice Is Highly Polyclonal and Dynamic DNA extracted from *in vitro* cultures, peripheral blood at 16 weeks, and bone marrow at termination was analyzed for vector integration. (A) Venn diagram showing the overlapping ISs between PB and BM. (B) Shannon diversity index values (y axis) in *in vitro* samples, PB, and BM. (C) Histograms of the percentage of sequence reads (y axis) for unequivocally mapped ISs from PB. The number of reads for each IS is normalized to the total number of sequence reads from the same time point and for the engraftment level. ISs below 1% were collapsed and are shown in gray. (D) ISs in BM.

Vector-genome junctions were retrieved by means of linear amplification-mediated PCR (LAM-PCR) by using three different restriction enzymes, as described previously.^3^, ^4^ To identify the vector ISs, the sequences of the PCR products generated by MiSeq Illumina sequencing were mapped on the mouse genome by a dedicated bioinformatics pipeline.^41^ Overall, we mapped 1,673 ISs from *in vitro* cultures, 3,080 from PB (at 16 weeks), and 1,738 BM cells (at 12 weeks). Tables S2A and S2B summarize the number of ISs identified for each sample. For the next set of biological analyses, we applied a threshold for the minimum number of reads for each IS (n = 3) with the goal to minimize the number of false positives. To study the clonal dynamics of the transduced cells during the hematopoietic reconstitution, we evaluated the sharing degree of ISs between the PB at 16 weeks and BM 1 year after transplantation and found that 30% of PB ISs were shared with the ISs in BM, indicating self-renewal and multilineage potential of the transduced engrafted HSPCs (Figure 2A).

Moreover, to quantify clonal diversity over time, we calculated the Shannon diversity index. This index measures the entropy of an IS dataset, taking into account the total number of ISs and their relative contribution. The diversity of ISs in BM 12 months after transplantation was lower compared with that of PB at 4 months in almost all sample analyzed, suggesting that a diverse clonal repertoire is established after transplantation with different active gene-corrected long-term (LT)-HSC contributing to hematopoiesis (Figure 2B).

To better investigate this aspect, we measured the proportion of sequencing reads representing each IS within our datasets as a surrogate readout for the relative abundance of a cell clone harboring that integration at a given time. In PB at 16 weeks, this analysis identified 1–2 clones in 70% of the analyzed mice with a relative abundance above 10%, a threshold arbitrarily set for defining dominant clones (Figure S4A). Only one mouse (952) harbored 2 integrations above 30% (Figure S4A). Thus, PB at 16 weeks showed a highly polyclonal reconstitution pattern.

At termination, in the BM, we found a less polyclonal pattern, with all mice harboring at least one clone with relative abundance above 10% (Figure S4B). Mouse 627 was of particular interest because we retrieved only 2 ISs, and one of them (targeting the Wdr78 gene) has a relative abundance of 99% in the BM, whereas it was at a very low level in PB at 16 weeks (Figure S4). The engraftment and VCN in this mouse were very low at both time points (Table S2B), indicating that few transduced clones are participating to host recovered hematopoiesis at low levels (Figures 2C and 2D). Furthermore, almost every cell clone marked by a specific IS accounted for only a fraction of a percentage of the total hematopoietic clones at any given time (Figures 2C and 2D).

We also analyzed the genomic distribution of ISs and the gene classes preferentially hit by LV integration. The genomic distribution of ISs, both in the *in vitro*-cultured Lin^−^ cells and *in vivo*, matched the previously reported LV preference for integration within transcriptional units (on average, 80% of ISs were within genes).^42^ In all groups, ≈80% of ISs were found between 5 and 500 kb from transcription start sites (Figures S5A–S5C). From these analyses, it is evident that the hematopoietic reconstitution of the mice transplanted with transduced cells is highly polyclonal and dynamic and that GLOBE LV shows the classical integration pattern characteristic of HIV-1-derived vectors.

**Figure 3:**
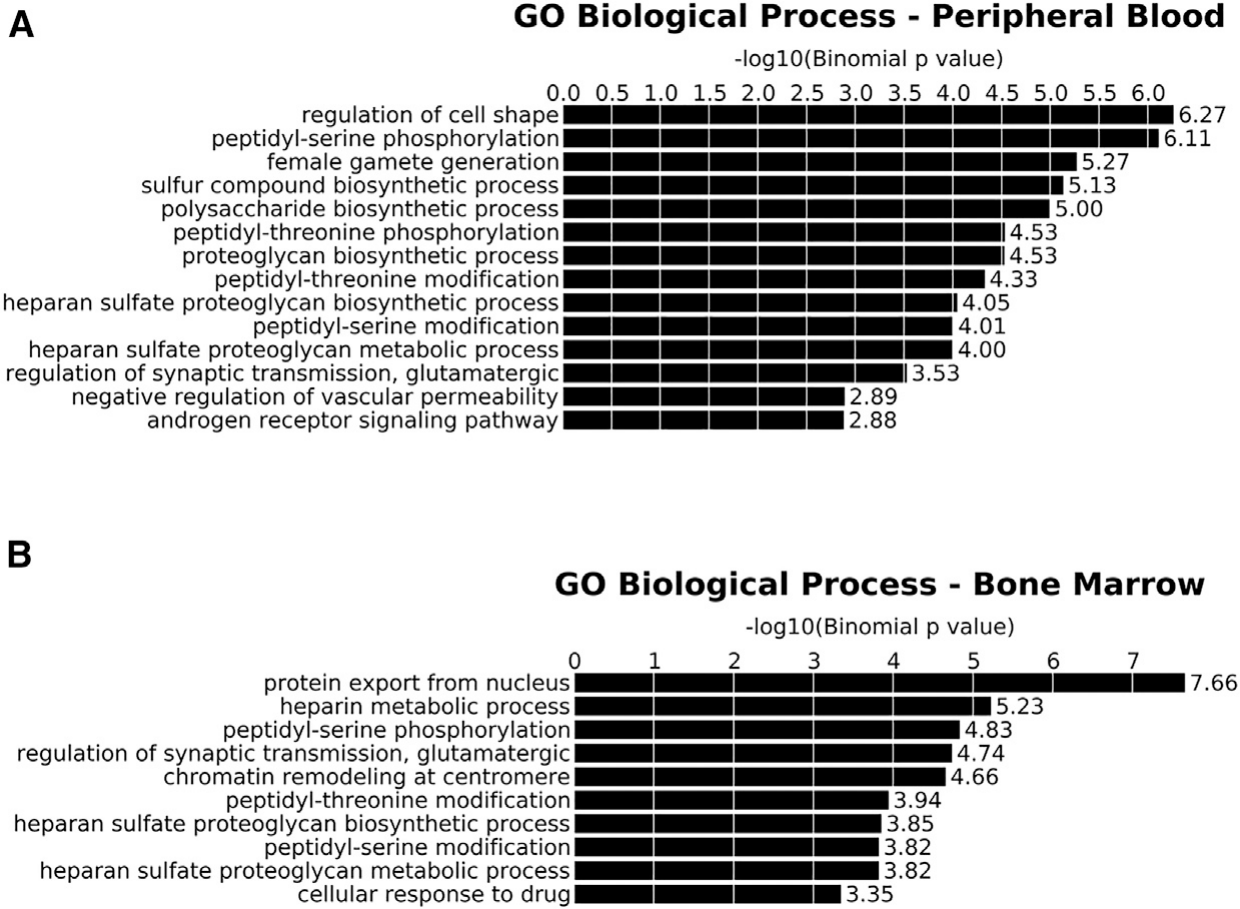
Gene Ontology Analysis of ISs Gene ontology (GO) analysis was performed using the GREAT software on *in vitro* cultures, peripheral blood at 16 weeks, and bone marrow at termination at 12 weeks. The integrations retrieved from each group showed overrepresentations of the gene functions indicated on the left. Only gene classes with a false discovery rate of less than 0.05 were considered. We interrogated the Biological Process database in (A) peripheral blood and (B) bone marrow.

To assess to which groups of biological processes genes with integrated vector belong, gene ontology (GO) enrichment analysis was performed. Interestingly, in *in vivo* data, we observed an enrichment in categories belonging to metabolic and biosynthetic processes (Figures 3A and 3B).

### Development of Lymphoproliferative Tumors Occurred Independently from GLOBE LV Integration

**Figure 4:**
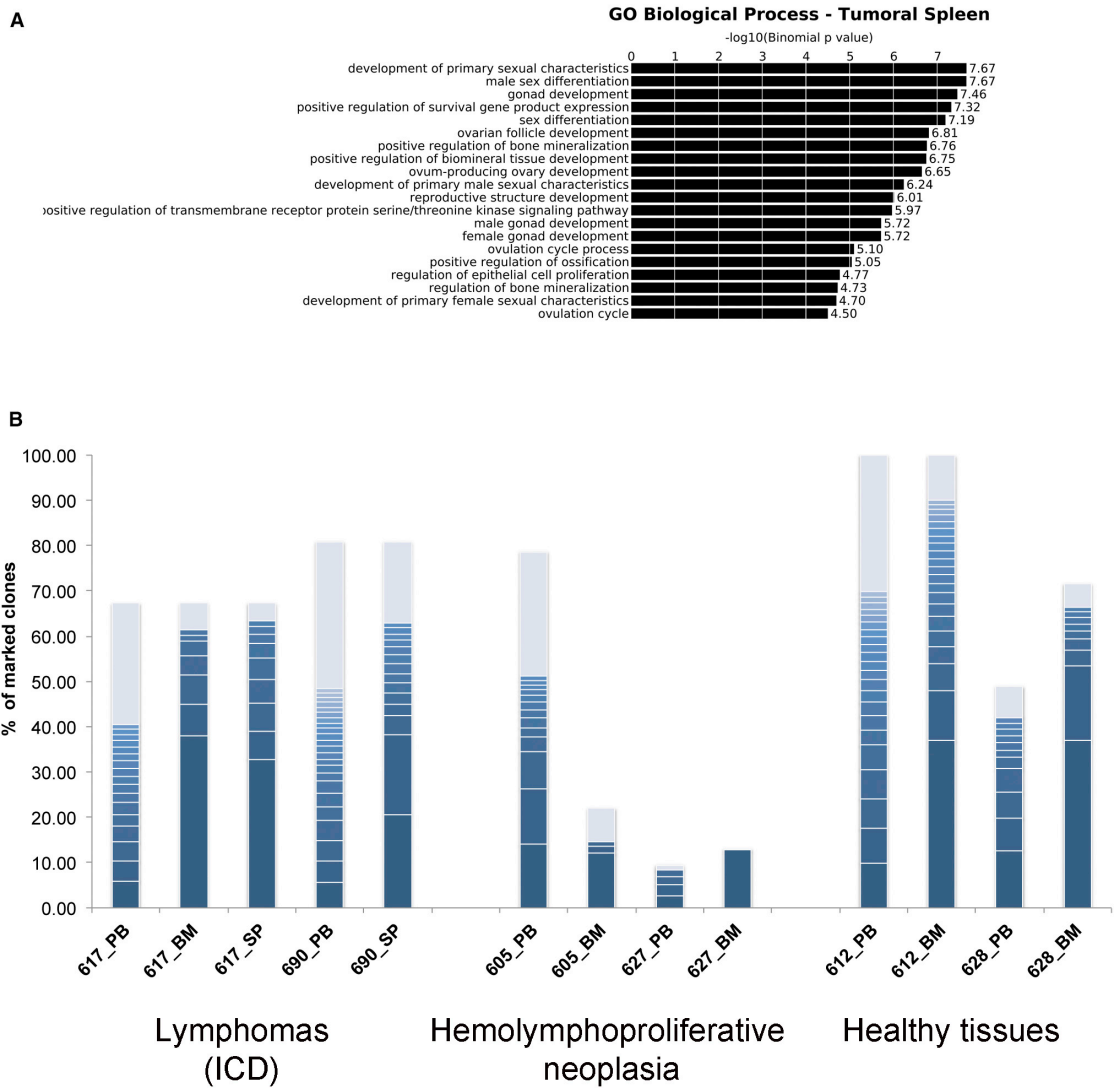
Gene Ontology Analysis and Abundance Analysis of ISs from Infiltrated Tumors DNA extracted from infiltrated tumor spleens was analyzed for vector integration (mice 617 and 690). (A) GO analysis was performed using the GREAT software. The integrations retrieved from tumors showed overrepresentations of the gene functions indicated on the left. Only gene classes with a false discovery rate of less than 0.05 were considered. We interrogated the Biological Process database. (B) Histograms of the percentage of sequence reads (y axis) for unequivocally mapped ISs from mice that developed lymphomas and hemolymphoproliferative neoplasia and healthy controls. The number of reads for each IS was normalized to the total number of sequence reads from the same time point and for the engraftment level. ISs below 1% were collapsed and are shown in gray.

To better define the biosafety of GLOBE LV, we completed IS analysis mapping in the infiltrated spleens of the two mice dead from lymphoma (617 and 690; Table 2). No differences in genomic distribution were detected with respect to the other IS groups analyzed (*in vitro*, PB, and BM) (Figure S5D), and GO (GO) analysis did not identify enrichment in terms belonging to cancer, abnormal proliferation, or similar, but it identified a significant over-representation of the sexual development categories (Figure 4A). In these mice, VCN analysis in lymphoma-infiltrated spleens showed vector marking at a lower level compared with the other hematopoietic tissues (Table 2), with a high donor cell chimerism in the blood (67% and 81%, respectively). Hemolymphoproliferative neoplasia was identified in two other mice (605 with histiocytic sarcoma and 627 with lymphoma) at termination.

Animal 617 was found dead and showed enlargement and discoloration of the spleen, thymus, mesenteric lymph nodes, and kidney. Samples of the spleen and thymus were frozen, and IHC was performed. A severe infiltration of atypical enlarged cells involving red and white pulp was seen. Infiltrating cells were positive for staining with anti-Ki67, CD3, and CD45.1 antibodies, suggesting a T origin of the lymphoma cells from donor-derived cells (data not shown). In the spleen of mouse 617, we identified one clone with a relative abundance of 48.8% (Figure S6). This IS was also retrieved in the BM at a higher relative abundance level (56.4%), whereas, at an earlier time point, it was at a very low level (2.8%) (Figure S6). Considering the correction for engraftment level, this clone accounted for 32.8%–37.9% of total hematopoiesis. The integration targeting a gene (Klra21) is not cancer-related; thus, it indicates that the tumor is marked by a neutral integration. Similar results were obtained in mouse 690, where we identified two ISs with a relative abundance of 25.5% and 21.7% that, considering the engraftment level, accounted for the ≈20% of total hematopoiesis (Figure 4B). Comparing these results with those in tumor-free mice, the clonal expansion was not relevant for cancer development (Figure 4B). Moreover, mice bearing hemolymphoproliferative tumors recorded at termination (605 and 627) showed dominant clones with lower abundance (≈12% in BM), whereas tumor-free mice had clones with a similar or higher abundance (≈37% in BM) (Figure 4B).

Then, we performed an analysis of cancer-associated genes using the Retroviral Tagged Cancer Gene Database (RTCGD) database. In all four samples (*in vitro*, BM, PB, and SP samples), ≈20% of genes are present in the database (Table S4). From the analysis of tumors, we did not see any enrichment in the abundance of integrations targeting cancer genes, suggesting that the integrations are not involved in the transformation process.

To determine whether other hallmarks of insertional mutagenesis were present in the GLOBE LV integration profile, we assessed the occurrence of common ISs (CISs), insertional hotspots that may result from integration bias at the time of transduction or *in vivo* selection of clones harboring integrations that confer a growth advantage. CISs were identified with an algorithm based on Abel et al.^43^ and by the Grubbs test for outliers.^34^ CIS analysis did not show any alarming sign of genotoxicity because the only significant CIS was found in the control dataset of *in vitro*-cultured cells. A less stringent correction of the p values obtained in our CIS analysis also allows finding “weaker” CISs in the integration datasets *in vivo* (PB and BM). Interestingly, the CISs found in BM 1 year after transplant are less and are targeted by fewer integrations than the CIS identified in PB 16 weeks after transplantation. Thus, even a less stringent correction of the CIS significance does not show any abnormal enrichment of CISs over time. Moreover, CISs were not represented by high sequence counts and did not preferentially target oncogenes.

Overall, these analyses showed a safe integration profile of GLOBE LV in murine HSC and that GLOBE LV is not directly linked to tumor development.

### Human CD34^+^ Cells Transduced with GLOBE LV Are Able to Engraft and Reconstitute NSG Mice

As a further step, we assessed whether the distribution of GLOBE genetically modified human CD34^+^ cells in the organs of the immunocompromised NSG mouse model was comparable with that of unmodified cells. We tested GLOBE LV-transduced human CD34^+^ cells for their ability to engraft, differentiate into lineage-specific progeny, and localize into lympho-hematopoietic murine tissues.

**Figure 5:**
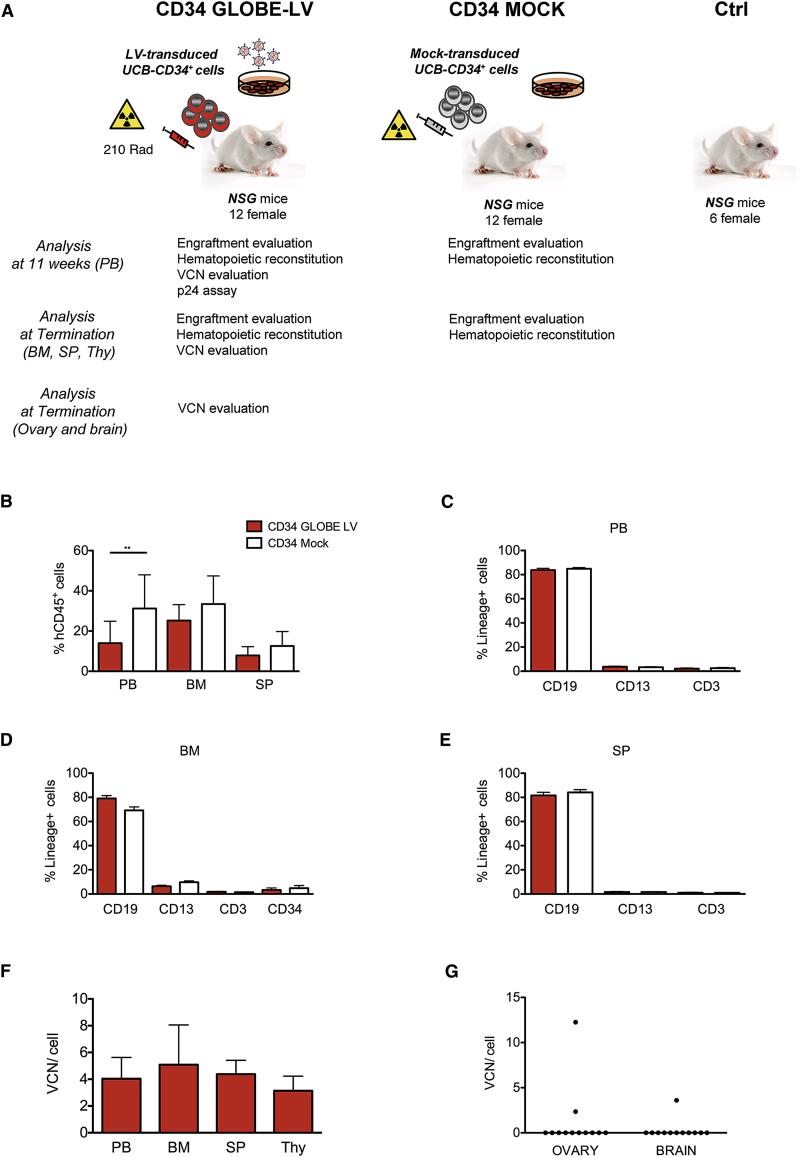
Biodistribution Study in the Human Chimera Murine Model (A) Experimental plan of the biodistribution study. Female NSG (NOD.Cg-Prkdcsid Il2rgtm1Wjl/SzJ) recipient mice were conditioned by a sub-lethal dose of irradiation to provide depletion of endogenous BM cells, allowing engraftment of human cells. After conditioning, NSG GLOBE LV mice (n = 12) were intravenously injected with transduced CD34^+^ cells, NSG MOCK mice (n = 12) were treated with MOCK-transduced cells, and NSG control mice (n = 6) were left untreated and served as controls. At the end of the study, the engraftment, the differentiation to specific lineages, and the persistence of transduced human cells in hematopoietic organs were evaluated. After extensive perfusion to eliminate contaminating blood, gonads and brain were analyzed by qPCR to evaluate the distribution of human cells in non-hematopoietic organs. HIV p24 was assayed in mouse serum to test for the absence of replication-competent lentivirus. (B) Analysis of hematological reconstitution was assessed by cytofluorimetric analysis in PB, BM, and SP by using anti-human CD45^+^ (**p < 0.01, two-tailed Student’s t test ). (C–E) PB (C), BM (D), and SP (E) showed multi-lineage differentiation of lymphoid and myeloid cells (CD19^+^, CD3^+^, and CD13^+^) both in transduced and MOCK-transduced cells. (F) VCN per cell was determined in PB, BM, SP, and thymus by qPCR. (G) VCN per cell was determined in ovaries and brain. All values are shown as mean ± SD.

Cord blood-derived CD34^+^ cells (UCB-CD34^+^) were transduced with a GLOBE LV batch manufactured under good manufacturing practice (GMP) conditions. Transduced (CD34 GLOBE LV) or MOCK-transduced (CD34 MOCK) cells were injected intravenously (i.v.) into sub-lethally irradiated NSG mice (CD34 GLOBE LV, n = 12; CD34 MOCK, n = 12). A third group of age-matched mice was included in the study as an untreated control group (CD34 Ctrl, n = 6). The mice were monitored daily for survival and weekly for clinical signs and body weight until 12 weeks after transplantation. At the end of the study, the engraftment, differentiation to specific lineages, and persistence of transduced human cells in hematopoietic organs (blood, BM, spleen, and thymus) were assessed. After extensive perfusion to reduce contaminating blood, gonads and brain were analyzed by qPCR to evaluate the distribution of human cells also in non-hematopoietic off-target organs (Figure 5A). There were no clinical signs considered to be related to the treatment and no mortality was observed during the study up to termination, except for one mouse in the CD34 MOCK group that died after bleeding at 11 weeks (day 77).

The immunophenotype analysis data showed successful and robust engraftment of transduced and MOCK-transduced UCB-CD34^+^ cells in NSG mice after sub-lethal irradiation. Statistical analysis showed a lower percentage of human CD45^+^ cells detected in the periphery in CD34 GLOBE LV mice (mean ± SD, 13.96 ± 3.146) compared with CD34 MOCK mice (31.24 ± 4.83) (p = 0.0066); however, no difference was found in the BM (25.23 ± 2.28 versus 33.40 ± 4.22) and in the spleen (17.86 ± 4.34 versus 12.56 ± 7.23) (Figure 5B). As expected, in the NSG model,^44^ the higher proportion of human cells was represented by B cells (CD19^+^ cells) in the PB and in the hematopoietic organs (Figures 5C–5E). The BM showed human HSPC engraftment (immunostaining with anti-human CD34) with multi-lineage differentiation of lymphoid and myeloid cells (immunostaining with anti-human CD19, CD3, and CD11b). No significant differences in CD34^+^ levels were observed between groups in the BM, indicating a comparable capacity of CD34 GLOBE LV cells (3.25 ± 0.50) and CD34 MOCK cells (4.76 ± 0.66) to engraft and reconstitute *in vivo* (Figure 5D).

No differences in VCN were observed among the target organs PB, BM, and SP (4.04 ± 1.59, 5.09 ± 2.97, and 4.38 ± 1.03, respectively) (Figure 5F). The average VCN measured in all examined hematopoietic tissue was comparable with the VCN measured in the bulk population of the transduced cells prior to administration (4.11).

At termination, non-hematopoietic organs (ovaries and brain) were collected from NSG mice after perfusion to assess the risk of germline transmission through vector shedding and secondary transduction or inadvertently generated replication-competent lentivirus (RCL). The ovaries of 10 of 12 mice in the CD34 GLOBE LV group were negative or below the LOQ (low limit of quantification) for VCN analysis (Figure 5G). In the ovaries of the other 2 mice, less than 0.5% of human cells were detected. The VCN of 12.26 in the ovary sample from mouse 1-05 was generated by a ratio between low LV copies and an amount of human DNA very close to the LOQ. The brains of 11 of 12 mice were negative or below the LOQ for VCN analysis (Figure 5G). The VCN of 3.60 in mouse 1-11 was below the PB VCN of the same mouse (6.43). These data are likely were likely caused by false positives or related to incomplete perfusion so that residual blood containing high VCNs can be measured in these samples, as shown in Visigalli et al.^45^ Calculation of VCN in mouse tissues, as a ratio between murine genomes (β-actin target ng) and LV genomes (HIV ng), excluded the association of LV with murine cells (data not shown). Occasionally, a low signal for vector DNA was detected in non-target organs of control group mice, setting the background threshold of sample processing and the qPCR procedure.

Serum samples from CD34 GLOBE LV mice (1-01, 1-02, 1-05, 1-08, and 1-09) that showed a low level of LV DNA in ovary and/or brain in the presence of low or undetectable levels of human DNA (hTELO ng) were also analyzed by HIV p24 assay to exclude the presence of circulating aberrantly generated RCL. All analyzed samples were negative in the HIV p24 detection assay; i.e., below the analytical sensitivity of the ELISA test (<3.125 pg/mL).

Overall, these results showed that the GLOBE medicinal product successfully engrafts and differentiates into the myeloid and lymphoid progeny at a level comparable with that observed for MOCK-transduced CD34^+^ cells. Notably, the proportion of human cells retaining the primitive CD45^+^/CD34^+^ phenotype was comparable in mice receiving GLOBE LV-transduced and MOCK-transduced cells. No evidence of LV transfer in non-target organs, gonads and brain, was found.

### *In Vitro* and *Ex Vivo* ISs Profiles of GLOBE LV in Human CD34^+^ Cells

**Figure 6:**
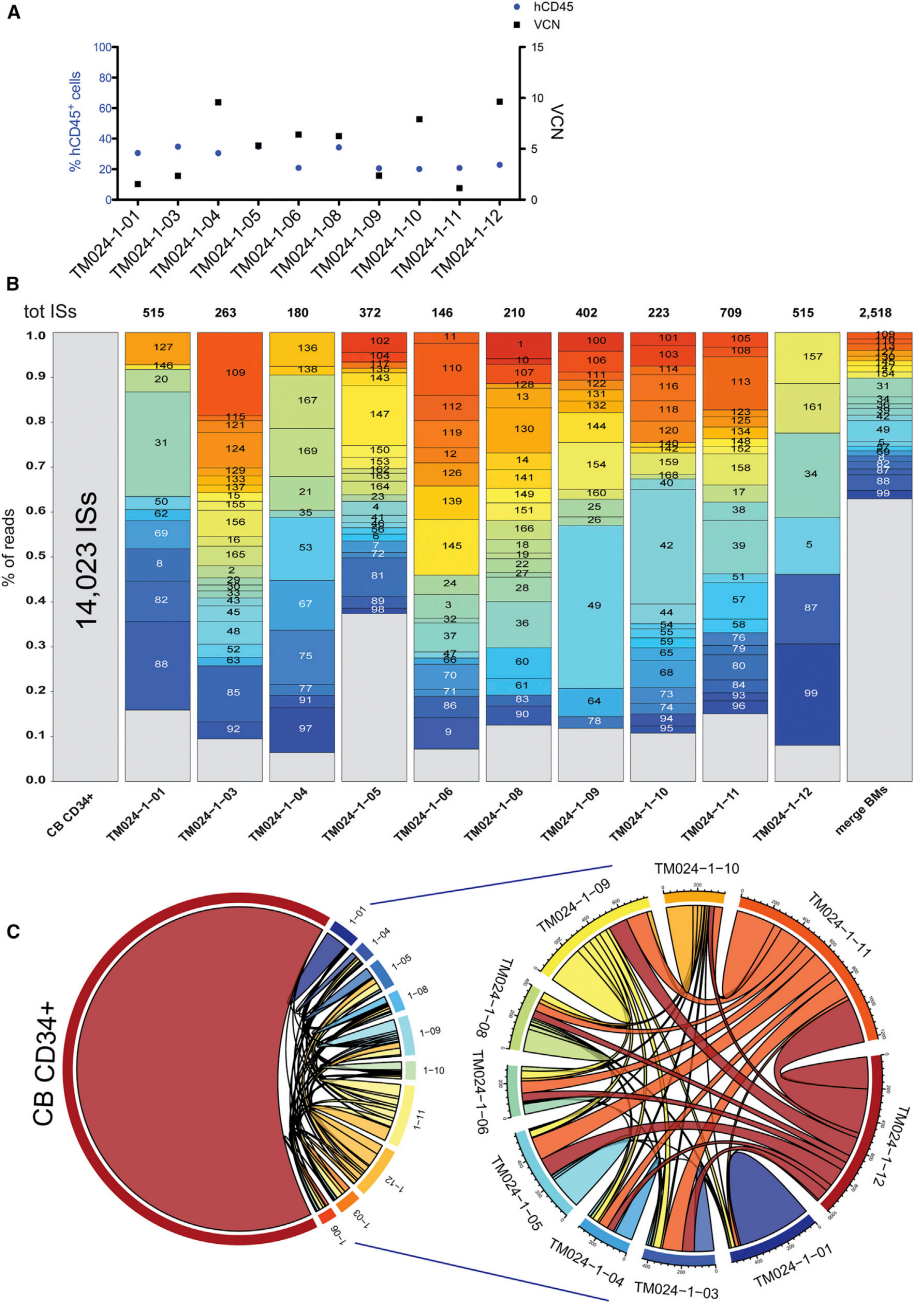
*In Vitro* and *Ex Vivo* Integration Site Analysis of LV GLOBE in Human HSPCs (A) Human versus mouse chimerism and VCN evaluation in the BM of transplanted NSG mice at the end of the study. (B) Read count distribution per single IS obtained in pre-transplant transduced CB CD34^+^ cells and in the different BMs of transplanted mice at the end of the study. The gray color indicates a read count lower than 1% of the total trimmed reads, whereas the other colors indicate a read count higher than 1%. ID numbers for each IS are indicated in the boxes. In the study, no ISs with a read count greater than 1% were shared among the individual transplanted mice. (C) Shown on the left and right, respectively, are chord diagrams indicating the shared ISs among the different samples, including or not including the pre-transplant CB CD34^+^ cells.

The GLOBE LV integration profile was determined by LM-PCR coupled to high-throughput sequencing *in vitro* in the pool of transduced human CD34^+^ cells before transplantation and *ex vivo* in the BM of the 10 transplanted NSG mice at the end of the study. All mice were injected with 0.5 × 10^6^ transduced CD34^+^ cells, with a resulting chimerism of 20%–35% human cells and a VCN ranging from 1 to 10 (Figure 6A). Overall, 14,023 unique LV ISs were retrieved in the transduced CD34^+^ cells and 2,518 ISs in the pool of transplanted mice at the end of the study, indicating a highly polyclonal BM repopulation. The LV integration profile was also determined in individual animals, retrieving an average of 354 ISs/mouse (range, 146–709 ISs; SD = 183; Table S5).

Overall, no or few dominant clones (i.e., accounting for 10% or more of the total reads) were evidenced in post-transplant libraries by analyzing the final ISs read count (Figure 6B). In single-mouse libraries, most of the ISs had a read count of lower than 1% (low frequency, shown in gray in Figure 6B), whereas a variable number of ISs (6–22) had a relatively higher frequency (more than 1%, colored boxes in Figure 6Bb), most likely reflecting the limited number of long-term repopulating hematopoietic stem cells in the transplanted BM. Altogether, ISs with a read count of more than 1% in any individual animal targeted 128 genes, which were not significantly enriched for any biological function by GO Database for Annotation, Visualization and Integrated Discovery (DAVID) analysis (data not shown). Interestingly, none of these ISs was found to be in common among the different animals.

The relative distribution of ISs inside and outside of genes changed in the post-transplant samples with respect to pre-transplant-transduced CD34^+^, with a significant increase in the fraction of intergenic ISs (χ^2^ test p < 0.0001). This effect was particularly evident in two of ten mice analyzed (TM024-1-01 and TM024-1-05; Table S5; Figure S8).

Overall, we retrieved 4,802 and 1,316 genes targeted by LV GLOBE in pre-transplant CD34^+^ cells and post-transplant BM, respectively. The number of targeted genes was positively correlated with the number of ISs (Figure S7A), with most of the targeted genes hosting 1 provirus (data not shown), ranging from 1–50 ISs/gene in pre-transplant CD34^+^ cells (50 ISs in PACS1, all low-frequency ISs), 1–12 ISs/gene in merged BM samples (12 ISs in LOC440434, all low-frequency ISs), and 1–9 ISs in individual mice. Among the top 100 genes targeted *ex vivo*, we found genes preferentially targeted by LVs, such as PACS1, KDM2A, and others, as observed in the pre-transplant CD34^+^ cells (Figure S7C) and in three LV-based clinical trials.^3^, ^46^ The genes targeted in pre-transplant CD34^+^ cells were significantly enriched in several biological functions (Figure S7D). Targeted genes in post-transplant BM were not significantly enriched in any GO biological function category or molecular pathway (Kyoto Encyclopedia of Genes and Genomes [KEGG] pathways dataset) with respect to the pre-transplant sample by DAVID analysis (data not shown), suggesting no selection for clones carrying LV integration in genes involved in particular functions. Although most of the targeted genes were in common in post- versus pre-transplant cells (Table S6), only 1 or 2 individual ISs were shared between pre- and post-transplant samples (Figure 6C; Table S7). On the contrary, several ISs were shared among individual mice (Figure 6C; Table S7), ranging from 8% to 64% of the total ISs (Figure S8).

## Discussion

Here we analyzed transgene expression and production from three different globin LVs, specifically GLOBE, GATA-GLOBE, and cHS4-GLOBE, and their potential deregulation of cellular genes located in the vicinity of or containing the vector insertion in human cells. No significant differences were observed among LVs. On the basis of these experiments and given the long development and deep characterization of the GLOBE LV,^22^ we decided to continue the safety characterization with GLOBE LV.

We observed that GATA-GLOBE LV produced more β-globin transcript than GLOBE LV. This higher transcription activity is due the presence of the GATA1-HS2 element in the LV, which binds the transcription factors CP2 and BCL11A.^47^, ^48^ Our data confirmed the potential GATA1-HS2 anti-silencing effect.^37^ However, this transcriptional difference was not confirmed at the protein level. Moreover, evaluation of the perturbation of genes hit by LV integration highlights that GLOBE LV deregulated gene expression with a frequency similar to the other β-globin-expressing LVs reported in this paper and in the literature.^18^, ^19^, ^20^ Hargrove et al.^20^ showed that vector integration perturbed the expression of genes with a frequency of 11%, whereas GLOBE LV showed a frequency of 4% and 7% in immature and more mature erythroid cells, respectively (Table S1).

The safety and efficacy of GLOBE LV were tested *in vivo* by transplantation experiments following the guidelines for GLP in the GLP-SR-Tiget test facility. The preclinical study for gene therapy of mucopolysaccharidosis I (MPS I), recently published by Visigalli et al.,^45^ was performed in this facility following the same guidelines. We designed an *in vivo* study aimed to evaluate the safety and efficacy of GLOBE LV in a mouse model of β-thalassemia intermedia (th3/+). Engraftment and transduction were very efficient, and the thalassemic phenotype was fully corrected in all mice, with two exceptions (620 and 952). Mouse 620 had very low engraftment (5.4%). Mouse 952 had an engraftment of 11.5% with a VCN in the marked cells of 1.39 (VCN 0.16 on total BM) and the most abundant clone, representing about 5% of total hematopoiesis. However, 2 mice with similar abundant clones were fully corrected with similar VCN in the marked cells (1.88–1.43). These data suggest that, in the presence of genomic low VCN per cell, the transcriptional activity of the vector might be influenced by surrounding chromatin.^49^ Interestingly, GLOBE LV corrects the thalassemia phenotype with 1 copy present in about 10% of total hematopoietic cells, as shown by mouse 627, confirming previously published data.^26^

VCN per-cell values were lower and with a higher variability in the female groups, a reflection of variability in engraftment level. This finding was not vector-related because it also occurred at a higher level in the control group (th3/+ MOCK mice). Variability of engraftment might be expected because of variable bioavailability of busulfan. Pharmacokinetic studies have verified great inter-patient variations because of age, circadian rhythmicity, and underlying diseases.^50^, ^51^, ^52^, ^53^ As an additional factor to this variability, a gender-related different liver metabolism, influencing drug clearance in the th3/+ strain, which is characterized by hepatic iron imbalance, can be hypothesized. A higher liver iron concentration in th3/+ females^54^ and a testosterone-related effect on iron homeostasis might influence metabolic liver function. Because dose adjustment, based on busulfan plasmatic dosage, is not feasible in mice, variable drug effects might have occurred, and a partial level of myeloablation because of biological variability and different sensitivity to busulfan treatment in females compared with males might have affected the level of engraftment of transplanted cells. Histopathological evaluation of tissues, clinical chemistry, and hematology did not reveal any vector-related safety concerns.

In the gene therapy-treated group, we reported 2 premature deaths because of generalized lymphoma and 2 mice with lymphoma and histiocytic sarcoma at terminal sacrifice. To understand whether these tumors could have been induced by insertional mutagenesis, the average VCN per cell was assessed in the spleens of premature decedent animals (617 and 690). These animals showed high donor cell chimerism (67% and 81%) and vector marking (12.92 and 15.87) in the blood but a lower VCN (≈2-fold) in the affected tissues (8.69 and 7.82). Moreover, because these malignancies were also present in the MOCK group (1 premature death and 1 terminal kill) and in the untreated group (5 terminal kills), they are not considered a treatment-related finding. A total of 8,362,036 sequence reads resulted from the appropriate data filtering process of the original reads. The relative abundance of sequences representing each IS (a clonal mark) with respect to the total number of sequences provided an estimation of the relative abundance of a cell clone in the transduced population. The highest estimated values of the abundance of individual cell clones at early (16 weeks) or late (at termination) time points after transplant was lower than 4% of the entire dataset, which is below the threshold value generally associated with clonal dominance.^3^, ^4^ To complete the safety profile of GLOBE LV treatment, IS analyses were performed on neoplastic tissue, PB, BM, and pre-transplant samples. As expected, transplantation of a high amount number of lineage-negative mouse BM cells resulted in a polyclonal hematopoietic repopulation with preferential insertions (80% of ISs) in gene coding regions. These ISs do not show evidence of enrichment in growth- or cancer-related genes, and no CISs were retrieved in *in vitro* and *in vivo* samples.

Moreover, GLOBE LV does not show preferred integration in either *MDS1-EVI1* or *LMO2*, which were previously linked to malignant transformation in the gene therapy setting. In fact, integration of gamma retroviral vectors into these loci has been associated with tumor development in patients treated for chronic granulomatous disease^55^ or X chromosome-linked severe combined immunodeficiency and Wiskott Aldrich syndrome.^56^, ^57^, ^58^ Together, these data indicate prediction of a safe integration profile of GLOBE LV.

In addition, the tumors we observed are not likely to be driven by integrations in dangerous genes, and our data are supported by a number of other studies describing spontaneous lymphomas in aging mice and undergoing conditioning.^29^, ^32^, ^56^, ^59^, ^60^, ^61^, ^62^ In fact, lymphomas have been reported to be the most commonly observed spontaneous tumors^62^ in C57BL6 mice,^61^ the background of our disease model. Moreover, chemicals, retroviruses, or irradiation induces lymphoma and leukemia.^32^, ^63^, ^64^ These studies suggest that there are other risk factors for oncogenesis in hematopoietic gene therapy protocols besides integration of LVs in the genome. Moreover, our data are in agreement with the findings of Visigalli et al.^45^ In conclusion, overall phenotype correction of thalassemic mice was observed with GLOBE LV, with no alteration of BM homeostasis in the transplanted animals.

Because the experimental medicinal product is represented by human autologous transduced CD34^+^ cells, and there is limited clinical experience with the *ex vivo* use of LV, we designed assays to assess the biodistribution and the potential risk of vector shedding, mobilization, and germline transmission using the relevant human target cells in *in vivo* models. In particular, we assessed whether the distribution of the GLOBE medicinal product (GLOBE LV genetically modified human CD34^+^ cells) in the organs of the humanized mouse model was comparable with that of unmodified cells. Our results indicate that the clinical-grade GLOBE LV can transduce, with high efficiency, human CD34^+^ progenitors. The two-hits protocol allowed us to reach 4 VCN/cell with no detectable toxic effect on *in vivo* differentiation of CD34^+^ cells. It should be noted that the selected protocol is already adopted in LV-based trials for genetic diseases where no side effects have been reported so far.^3^, ^4^

Our results show that GLOBE LV-transduced HSCs migrate to the hemolymphopoietic organs and undergo *in vivo* short-term differentiation, similar to unmodified HSCs. Our data, combined with previous observations,^45^ indicate that the use of the immunodeficient strain NSG, which allows efficient engraftment of human cells, represents a suitable model for assessing the biodistribution of human CD34^+^ cells.^33^ Our data show that LV distributes along with human hematopoietic cells, remaining integrated in the human genome, and is not mobilized or shed to murine cells. In addition, HSPC with the integrated vector can be detected in several tissues, including germlines. The detection of HIV sequences in the ovaries and/or brain of 5 mice together with the absence of HIV p24 gag antigen in the serum excluded the possibility that an RCL may have been generated. The safety features of the third-generation packaging system combined with a SIN transfer vector used in this study make it extremely unlikely that a productive viral recombinant is generated during vector production or cell transduction. Thus, the most likely interpretation of the findings is an incomplete perfusion procedure, so that residual blood containing high VCNs and human cells also in the MOCK group can be measured in these mice. As in the murine study, we performed IS analysis of the infused product to determine the safety profile of the GLOBE LV medicinal product. The transduced CD34^+^ cells showed a high polyclonal pattern of integration with no expansion or selection of specific ISs. Moreover, the genes preferentially targeted by GLOBE LV were PACS1, KDM2A, and others, which are well known hotspots in preclinical studies of LVs^34^, ^65^ and in lentiviral gene therapy trials and have not been associated with clonal expansion.^3^, ^4^, ^46^ We were able to detect several ISs shared among individual mice, suggesting that our transduction protocol maintains engraftment potential and that hematopoietic primitive cells remain multipotent and are able to generate new blood cells.

In summary, our data demonstrate that the GMP-grade β-globin encoding GLOBE LV can efficiently transduce CD34^+^ cells under validated clinical conditions with no toxicity. These studies provide important information about the quality and the safety data of transduced HSCs for thalassemic patients, complementing previous *in vitro* and *in vivo* studies performed in the murine th3/+ model, which showed efficient engraftment of vector-transduced cells and immune reconstitution without the occurrence of LV-induced toxicity and tumorigenicity tumors. Thus, this work strongly supports the translation of our gene therapy approach to the clinic as a phase I and II gene therapy clinical protocols based on infusion of GLOBE LV-transduced autologous CD34^+^ cells. Our trial received approval from regulatory authorities and has recently started (ClinicalTrials.gov identifier NCT02453477).

## Materials and Methods

### LVs

The GLOBE and GATA-GLOBE LVs have been described previously.^26^, ^37^ The cHS4-GLOBE LV was generated by cloning a BamHI 1.2-kb fragment containing the HS4 element from the chicken genome^18^, ^66^ into SpeI-SfiI sites of the LTR of the GLOBE vector backbone.^26^

Research-grade viral vector stocks were prepared by transient co-transfection of HEK293T cells using a three-plasmid system as described previously.^26^

For toxicology and biodistribution studies, we derived the pCCL.GLOBE Kana LV from the original PRRL GLOBE LV described previously. The pRRLsincpptW1.6Wasp-WPREmut6-Kana (a kind gift from L. Naldini) provided the main backbone.

Medium- and large-scale purified vector lots for the GLP studies were produced by MolMed (Milan, Italy). Vectors were produced by a medium- or large-scale process based on transient quadri-transfection in 293T cells, followed by purification through endonuclease treatment, anion exchange chromatography with gradient elution, gel filtration, resuspension in serum-free medium, 0.22-μm filtration, and aseptic filling. Each lot was characterized in terms of titer, potency, purity, and safety aspects (titer of the vector in the toxicology study, 9.69 × 10^7^ TU/mL; titer of the vector in the biodistribution study, 6.6 × 10^8^ TU/mL).

### Transduction and Culture of Cell Lines

The HEL cell line was purchased from the ECACC (European Collection of Cell Cultures). HEL cells were grown in RPMI 1640 medium (Lonza, Basel, Switzerland) supplemented with 10% fetal bovine serum (FBS) (GE Healthcare Life Science, Little Chalfont, UK), 100 U/mL penicillin and streptomycin, and 2 mM L-glutamine (Lonza, Basel, Switzerland) and transduced with viral stocks at a multiplicity of infection (MOI) of 20 in the presence of 8 μg/mL Polybrene (Sigma-Aldrich, St. Louis, MO).

Terminal erythroid differentiation was obtained by culturing the cells in the presence of 50 μM hemin (Sigma-Aldrich, St. Louis, MO) for 5 days.

Single cell-derived clones were obtained by limiting dilution in 96-well plates (0.3 cells/well). Clones were screened by vector-specific PCR.

### Southern and Northern Blot Analyses

Genomic DNA was extracted using the QIAamp DNA blood mini kit (QIAGEN, Hilden, Germany). Ten micrograms of DNA were digested overnight with AflII, EcoRV, SpeI, XbaI, or KpnI (all from New England Biolabs, UK), run on a 0.8% agarose gel, transferred to a nylon membrane (Hybond-N; GE Healthcare Life Science, Little Chalfont, UK) by Southern capillary transfer, probed with 2 × 10^7^ dpm of a 32P-labeled HS3 probe, and exposed to X-ray film.

Total cellular RNA was extracted by TRI reagent (Sigma-Aldrich, St. Louis, MO), size-fractionated on 1% agarose-formaldehyde gel, blotted onto nylon membranes, hybridized to 10^7^ dpm of 32P-labeled GATA-1 and glyceraldehyde-3-phosphate dehydrogenase (GAPDH) probes, and exposed to X-ray film.

### Western Blot Analysis

Protein extraction was performed by resuspending cells in lysis buffer (150 mM NaCl, 1% v/v NP40, 0.5% w/v deoxycholic acid, 0,1% w/v SDS, and 50 mM Trizma base [pH 7.8]) and centrifuged to remove cellular debris. Five micrograms of protein were then mixed with NuPAGE LDS sample buffer and NuPAGE sample-reducing agent (Invitrogen, Carlsbad, CA), boiled, and separated by SDS-PAGE on a 10% polyacrylamide gel. After being transferred to nitrocellulose membranes, the blots were blocked with PBS containing 0.1% Tween 20 and 5% milk for 1 hr at room temperature. The blots were probed for 1 hr at room temperature with an appropriate dilution of the primary antibodies. After washing with PBS containing 0.1% Tween 20, horseradish peroxidase-conjugated anti-mouse immunoglobulin G (IgG) was applied to the membrane for 1 hr at room temperature. The signals were then visualized with an enhanced chemiluminescence (ECL) kit (GE Healthcare Life Science, Little Chalfont, UK), and the intensity of the bands was quantified by ImageJ software (https://imagej.nih.gov/ij/).

### Quantitative Analysis of Gene Expression

Total cellular RNA was extracted by TRI reagent (Sigma-Aldrich, St. Louis, MO), purified using the Purelink RNA mini kit (Invitrogen, Carlsbad, CA), and treated with DNase I (Sigma-Aldrich, St. Louis, MO) to remove DNA contaminations. The absence of genomic DNA was verified by genome-specific PCR. Reverse transcription was performed using a high-capacity cDNA reverse transcription kit (Applied Biosystems, Foster City, CA), following the manufacturer’s instructions.

Gene expression levels were evaluated on a custom low-density array card (“Assay on Demand,” Applied Biosystems, Foster City, CA) using an ABI PRISM 7900 HT system. Relative quantification was calculated using the ΔCt method.^67^ ΔCt was calculated for every gene as Ct _(gene)_ − average Ct _(GAPDH, β2 microglobulin, RPLP0)_. ΔΔCt was calculated for test clones as Ct_(test)_ − average Ct_(controls)_. The relative quantification (RQ) of each transcript was calculated as 2^–ΔΔCt^ and plotted as log_2_ values.

### Analysis of VCN

To evaluate the number of LV copies integrated per genome, qPCR was performed using specific primers and probes for human telomerase, murine β-actin, and a LV, as described previously.^33^ A reference standard was obtained from serially diluted transduced human T cell lines carrying one copy of the integrated LV or murine NIH 3T3 cell line carrying six copies of LV. The results of integrated vector copies were normalized for the number of evaluated genomes. As a negative control, samples of untransduced cells were used. All the reactions were performed according to the manufacturer’s instructions and analyzed with an ABI PRISM 7900 sequence detection system or ViiA7 real-time PCR system (Applied Biosystems, Foster City, CA).

### GLOBE IS Mapping and Analysis

ISs were cloned by LAM-PCR and LM-PCR.

For LAM-PCR, after linear PCR amplification of genome-vector junctions, followed by magnetic capture and second strand reconstitution, digestion with Tsp509I, HpyCH4IV, or MseI was performed. Digestion products were ligated with a restriction site-complementary linker cassette and underwent two sequential rounds of exponential PCR steps. The LAM-PCR products were tagged with megaprimers and underwent next-generation sequencing on the MiSeq instrument (Illumina). The resulting genomic sequences were automatically aligned to the mouse genome (July 2007 assembly mm9 of the University of California Santa Cruz (UCSC) genome browser; http://genome.ucsc.edu/).

The relative abundance of ISs was computed from the matrix files cleaned by contaminations (also called collisions). For each sample, the abundance value was obtained as the percentage for each IS of the sequence reads over the total reads of the samples. When applicable, abundance was also correct for engraftment as abundance/engraftment × 100.

All GO analyses were made using the Genomic Regions Enrichment of Annotations Tool (GREAT) online software (http://bejerano.stanford.edu/great/public/html/). We uploaded the genomic coordinates of the integrations of each dataset and calculated the enrichment levels in the tested dataset by correlating positional information (based on the binomial distribution analysis for p value calculations) and the annotated function of the genes closest to the ISs (based on the hypergeometric distribution analysis for p value calculations). Biological processes of the GO database were chosen for enrichment analysis. Only gene classes with a false discovery rate of less than 0.05 were considered.

For LM-PCR, 0.150–500 ng of genomic DNA was digested with the MseI restriction enzyme (Roche). The digestion products were purified (NucleoSpin Gel and PCR Clean-up, Macherey-Nagel) and ligated to a restriction site-complementary double-stranded DNA linker. The ligation products were purified and digested with the SacI restriction enzyme (Roche) to avoid amplification of the internal provirus. Vector-genome junctions were amplified by LM-PCR, a nested PCR performed with specific primers annealing on the linker and the 3′ viral LTR, containing a 4-bp tag for sample identification. Finally, LM-PCR libraries were quantified, size-selected by gel extraction, checked by capillary electrophoresis (2100 Bioanalyzer Instrument, Agilent Technologies), and tagged with sample-specific dual indexes and Illumina sequencing adapters (Nextera XT Index Kit, Illumina) before being sequenced to saturation on the MiSeq instrument (Illumina). The resulting genomic sequences were demultiplexed by sample-specific tag and bioinformatically processed, trimmed by Skewer^68^ and mapped on the human genome (February 2009, GRCh37/hg19) by Bowtie2 software.^69^ During the mapping step, a quality score was assigned to each insertion site based on the total number of trimmed reads collapsed in that genomic position (read count) and the mean read quality. For each sample, the abundance value was obtained as the percentage of the read count over the total reads of the samples. DAVID software^70^ was used for GO analysis LV-targeted genes (Refseq database), considered as transcriptional units with inside at least one IS.

### Mice, Murine HSPC Transduction, and Transplantation

C57BL/6-Hbb^th3^ CD45.2 mice were purchased by Jackson Laboratory. C57BL/6-Hbb^th3^ CD45.1 mice were generated in our institute. We bred C57BL/6 CD45.1 mice to C57BL/6-Hbb^th3^ CD45.2 mice and then intercrossed the C57BL/6 Hbb^th3^ CD45.1/CD45.2 progeny and C57BL/6 CD45.1 to generate C57BL/6-Hbb^th3^ CD45.1 mice. All procedures were performed according to protocols approved by the Committee for Animal Care and Use of the San Raffaele Scientific Institute (Committee Protocol 537).

Recipient C57BL/6-Hbb^th3^ Ly-5.1 mice were treated intraperitoneally with busulfan (Sigma-Aldrich, St. Louis, MO) at 25 mg/kg/day for 4 days to obtain myeloablation prior to transplantation. Prophylactic antibacterial therapy was given by administration of gentamycin sulfate 80 mg/252 mL (0.3 mg/mL) by water intake from day −7 to day +7. Donor C57BL/6-Hbb^th3^ mice were killed by CO_2_ lethal inhalation, and BM from both tibia and femora was harvested into Miltenyi Biotec buffer to obtain Lin^−^ HSPCs. Lin^−^ HSPCs were purified with a Lineage Cell Depletion Kit Mouse (Miltenyi Biotec, Auburn, CA), seeded in StemSpan medium (STEMCELL Technologies, Vancouver, BC, Canada) supplemented with mIL3 (10 ng/mL), mIL6 (20 ng/mL), mFLT3L (10 ng/mL), and murine stem cell factor (mSCF; 50 ng/mL) (all from PeproTech, Rocky Hill, NY). Lin^−^ HSPCs were transduced with GLOBE LV at MOI 100 for 16–18 hr. MOCK-transduced cells were cultured under the same conditions in the absence of GLOBE LV to prepare the control item.

Transduced or MOCK-transduced cells were injected (4 × 10^5^ cells per mouse) i.v. in 8- to 12-week-old C57BL6/Hbb^th3^ mice.

*In vitro* liquid culture of transduced or MOCK-transduced Lin^−^ HSPC progeny was performed to conduct VCN and IS analysis. 1 × 10^6^ Lin^−^ HPSCs were seeded in liquid culture medium supplemented with 5% FBS with mIL3 (10 ng/mL), mFLT3L (10 ng/mL), mIL6 (20 ng/mL), and mSCF (50 ng/mL) (all from PeproTech, Rocky Hill, NY) and cultured for 12 ± 2 days. 2.5 × 10^3^ transduced or MOCK-transduced Lin^−^ HPSCs were seeded in methylcellulose medium (MethoCult M3434; STEMCELL Technologies, Vancouver, BC, Canada), colonies were scored on day 10 ± 2, and DNA was prepared for VCN analysis.

### Post-transplant Monitoring

Mortality and morbidity were checked once a day each working day. Animals were observed individually once a week for the recording of clinical signs and body weight. Blood samples for hematology analyses and DNA analyses were collected 16 weeks after transplantation and at termination. Erythrocyte count, mean cell volume, packed cell volume, hemoglobin, mean cell hemoglobin concentration, mean cell hemoglobin, thrombocyte count, leucocyte count, differential white cell count with cell morphology, and reticulocyte count were determined using the Scil Vet ABC Plus^+^ analyzer (Scil Animal Care, Treviglio, Italy).

Cytofluorimetric analyses of PB were performed on a Becton Dickinson LSR II machine with BD FACSDiva software. PB from mice was analyzed for hematopoietic reconstitution using the following antibodies: phycoerythrin (PE)-conjugated anti-mouse CD11b, anti-mouse CD3, and anti-mouse B220 (BD Biosciences, San Jose, CA). Fluorescein isothiocyanate (FITC)-conjugated anti-mouse CD45.1 and allophycocyanin (APC)-conjugated anti-mouse CD45.2 antibodies (BD Biosciences, San Jose, CA) were used to evaluate donor-host chimerism at 16 weeks and termination.

Genomic DNA was extracted from the blood for determination of VCN at 16 weeks using the QIAamp DNA Mini Kit (QIAGEN, Hilden, Germany).

### Necropsy, Histology, and Pathology

Animals found dead during the study or sacrificed in poor condition were subjected to necropsy, and selected tissues were harvested and fixed in 10% neutral buffered formalin (Sigma-Aldrich, St. Louis, MO). At termination, animals were shipped to Accelera (Nerviano, Italy), where animals were sacrificed, and a full necropsy was executed in compliance with the Italian GLP Regulations (DL 50; March 2, 2007; G.U. 8, April 13, 2007) and the OECD Principles of GLP (as revised in 1997, ENV/MC/CHEM(98)17). Tissues were sent to GlaxoSmithKline Research and Development for histology processing, and pathological examination was performed in accordance with United Kingdom GLP (statutory instruments number 3106 and 2004 number 994) and the OECD Principles of GLP (as revised in 1997, ENV/MC/CHEM(98)17). In case of macroscopic lesions, a part of the tissue was embedded in optimal cutting temperature (O.C.T., Tissue-Tek Sakura) medium and frozen in liquid nitrogen for possible further characterization.

The following organs were collected: adrenal gland, brain including medulla and pons, cerebellar and cerebral cortex, epididymis, heart, kidneys, large intestine (cecum, colon, and rectum), liver, lungs with bronchi, lymph nodes (mesenteric and mandibular), ovaries with oviduct, pancreas, skeletal muscle, skin, small intestine (duodenum, ileum, and jejunum), spleen, sternum with BM, stomach with forestomach, testes, thymus, uterus, and vagina. Tissues were embedded in paraffin wax, sectioned, mounted on glass slides, and stained with H&E in accordance with GlaxoSmithKline (GSK) standard operation procedures (SOPs). Tissue reading was performed at GSK Research and Development in accordance with GSK SOPs. A histopathology peer review was carried out by a second pathologist in accordance with GLP SR-Tiget SOPs.

For morphological analysis, 3-μm cryosections were fixed with 4% paraformaldehyde and stained with H&E. For immunohistochemical analysis, 3-μm cryosections were fixed with 4% paraformaldehyde at room temperature and incubated with rat anti-mouse B220 (clone RA3-6B2, Serotec), anti-mouse CD3 (clone CD3-12, Serotec), anti-mouse F4/80 (clone CI, A3-1, AbD Serotec), and rabbit Ki67 (clone SP6, Lab Vision) antibodies. 3-μm cryosections were fixed with acetone at 4°C and incubated with biotin-conjugated anti-mouse CD45.1 (clone A20, BD Pharmingen) and with biotin-conjugated anti-mouse CD45.2 (clone 104, BD Pharmingen). The immunoreactions were revealed either by rat or rabbit on rodent HRP polymer (Biocare Medical) by HRP-conjugated streptavidin and using diaminobenzidine (DAB) as chromogen (Biogenex, San Ramon). Slides were counterstained with hematoxylin.

Approximately 500–800 μL of blood was collected from the aorta during necropsy for chemical analyses. Glucose, urea, creatinine, triglycerides, total protein, albumin, albumin-to-globulin ratio, globulin (calculated parameter), alkaline phosphatase, alanine aminotransferase, and aspartate aminotransferase were measured by Accelera.

### Human CD34^+^ Cell Transduction and Xenotransplantation

Cryopreserved human cord blood CD34^+^ cells were purchased from Lonza (Basel, Switzerland). Cells were thawed and seeded on non-tissue culture-treated plates coated with Retronectin (30 μg/mL; TaKaRa Bio, Shiga, Japan) () at 1 × 10^6^ cells/mL in serum-free CellGro stem cell growth medium (SCGM; Cell Genix Technologies, Freiburg, Germany) in the presence of hSCF (300 ng/mL), hFLT3-L (300 ng/mL), thrombopoietin (100 ng/mL), and interleukin-3 (IL-3; 60 ng/mL) (all from PeproTech, Rocky Hill, NY). Following 24 hr of pre-stimulation, cells were transduced twice with GLOBE LV at MOI 100.^71^ MOCK-transduced control cells were cultured under the same conditions except for the presence of LV.

For transplantation studies *in vivo*, 2 × 10^5^ transduced or MOCK-transduced cells were administered to NSG mice (Jackson Laboratory) by injection in the lateral tail vein. Mice were maintained in a specific pathogen-free animal facility. Procedures were performed according to protocols approved by the Committee for Animal Care and Use of San Raffaele Scientific Institute (Committee Protocol 537). 9- to 10-week-old NSG female mice were conditioned by a sub-lethal dose of irradiation to provide depletion of endogenous BM cells, allowing engraftment of human cells. The recipient mice were exposed to 210 cGy of total body irradiation in an X-ray Rad Source RS-2000 irradiator (Biological System). Prophylactic antibacterial therapy was given by administration of gentamycin sulfate 80 mg/252 mL (0.3 mg/mL) by water intake from day −1 to day +28.

### *Ex Vivo* Analysis of Human Cells in Murine Tissues

After 12 weeks, mice were anesthetized and perfused by injecting saline solution for approximately 30 min through blood vessels to eliminate hematopoietic contamination from organs before sacrifice. Mice were euthanized, and the BM, spleen, and thymus were collected and analyzed for the presence of human CD45^+^ cells and the expression of different lineage-specific surface markers. DNA extracted from cells and tissues was analyzed for VCN by qPCR.

### p24 Assay

An HIV p24 ELISA (PerkinElmer, code NEK050) was performed on serum (20–40 μL) of treated mice according to the manufacturer’s instructions.

### Statistical Analyses

GraphPad Prism was used to perform statistical analyses: one-way ANOVA, *post hoc* Bonferroni’s multiple comparisons test or Student’s t test, and two-tailed log rank Mantel-Cox test. A Z test for proportion was performed for specific analyses.

## Author Contributions

M.R.L. carried out experiments, analyzed the data, and wrote and reviewed the manuscript. Y.P. analyzed the data, helped with interpretation of the results, and wrote the manuscript. F.T., G.M., C.R., M.V., and A. Aprile performed experiments. C.L. carried out the gene expression analysis in HEL cells. A. Ambrosi performed the statistical analysis of gene expression data. F.C. and F.S. performed the histopathological analysis and reviewed the manuscript. A.C. and E.M. carried out the integration site sequencing analysis for the toxicology study. V.P. and F.M. carried out the integration analysis for the biodistribution study. L.N. contributed to discussion of the results. P.C. contributed to the histopathology report and reviewed the manuscript. G.F. provided financial support, designed experiments, interpreted results, and wrote and reviewed the manuscript.

## Conflicts of Interest

The authors have no conflicts of interest.

## Supplemental Information

Document S1. Figures S1–S8 and Tables S1–S7

Document S2. Article plus Supplemental Information
