# Supplementary material for: Multiple Integrated Non-clinical Studies Predict the Safety of Lentivirus-Mediated Gene Therapy for β-Thalassemia
Source: Mol Ther Methods Clin Dev. 2018 Sep 13;11:9–28. doi: 10.1016/j.omtm.2018.09.001 (PMC6178212; doi:10.1016/j.omtm.2018.09.001)
Supplement: Document S1. Figures S1–S8 and Tables S1–S7 [file mmc1.pdf]

OMTM, Volume *11*

Supplemental Information

Multiple Integrated Non-clinical Studies Predict the Safety of Lentivirus-Mediated Gene Therapy for β-Thalassemia

Maria Rosa Lidonnici, Ylenia Paleari, Francesca Tiboni, Giacomo Mandelli, Claudia Rossi, Michela Vezzoli, Annamaria Aprile, Carsten Werner Lederer, Alessandro Ambrosi, Franck Chanut, Francesca Sanvito, Andrea Calabria, Valentina Poletti, Fulvio Mavilio, Eugenio Montini, Luigi Naldini, Patrizia Cristofori, and Giuliana Ferrari

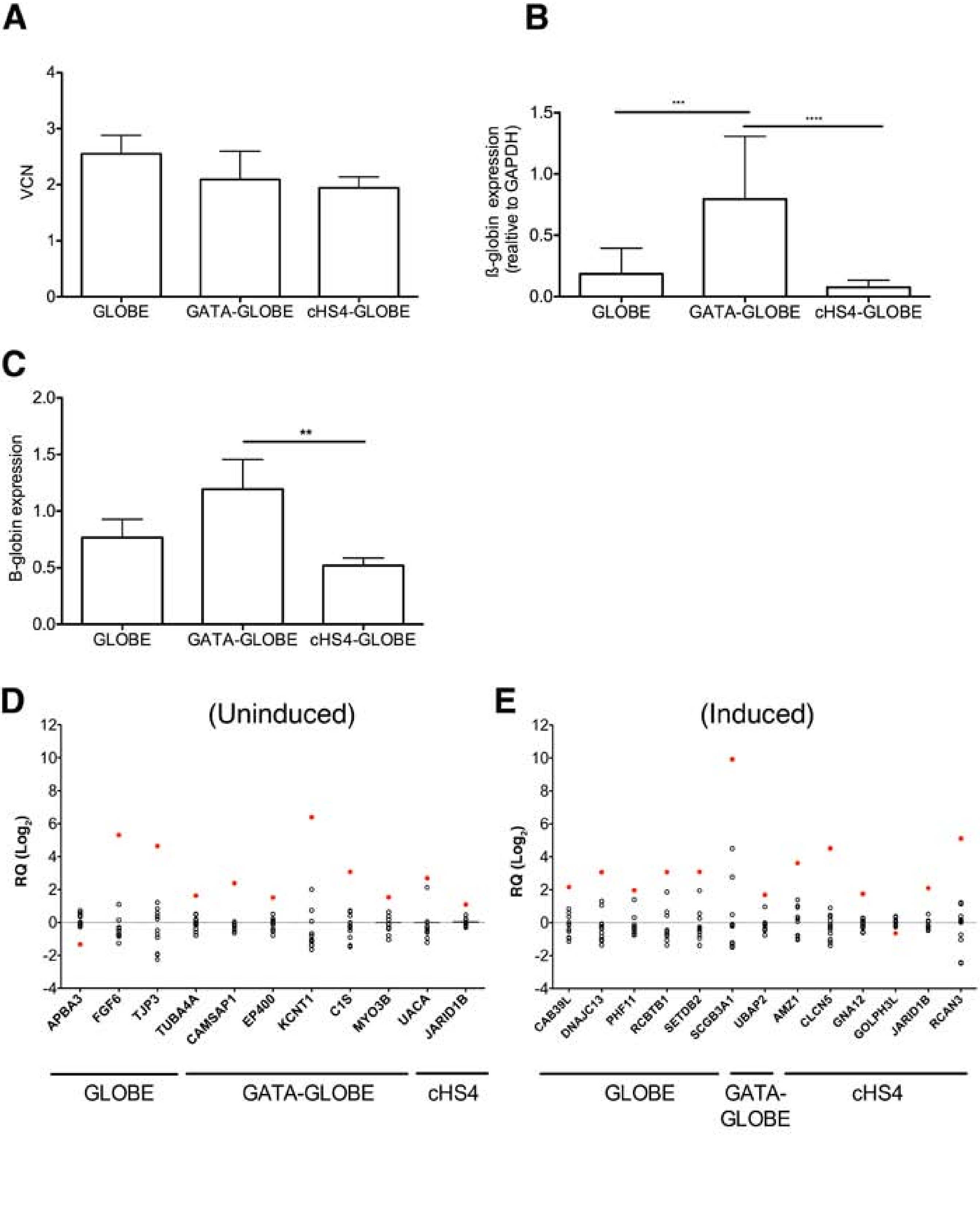

A
VCN
GLOBE
GATA-GLOBE
cHS4-GLOBE
B
ß-globin expression (realtive to GAPDH)
***
****
GLOBE
GATA-GLOBE
cHS4-GLOBE
C
B-globin expression
**
GLOBE
GATA-GLOBE
cHS4-GLOBE
D
(Uninduced)
RQ (Log2)
APBA3
FGF6
TJP3
TUBA4A
CAMSAP1
EP400
KCNT1
C1S
MYO3B
UACA
JARID1B
GLOBE
GATA-GLOBE
cHS4
E
(Induced)
RQ (Log2)
CAB39L
DNAJC13
PHF11
RCBTB1
SETDB2
SCGB3A1
UBAP2
AMZ1
CLCN5
GNA12
GOLPH3L
JARID1B
RCAN3
GLOBE
GATA-GLOBE
cHS4

Fig. S1

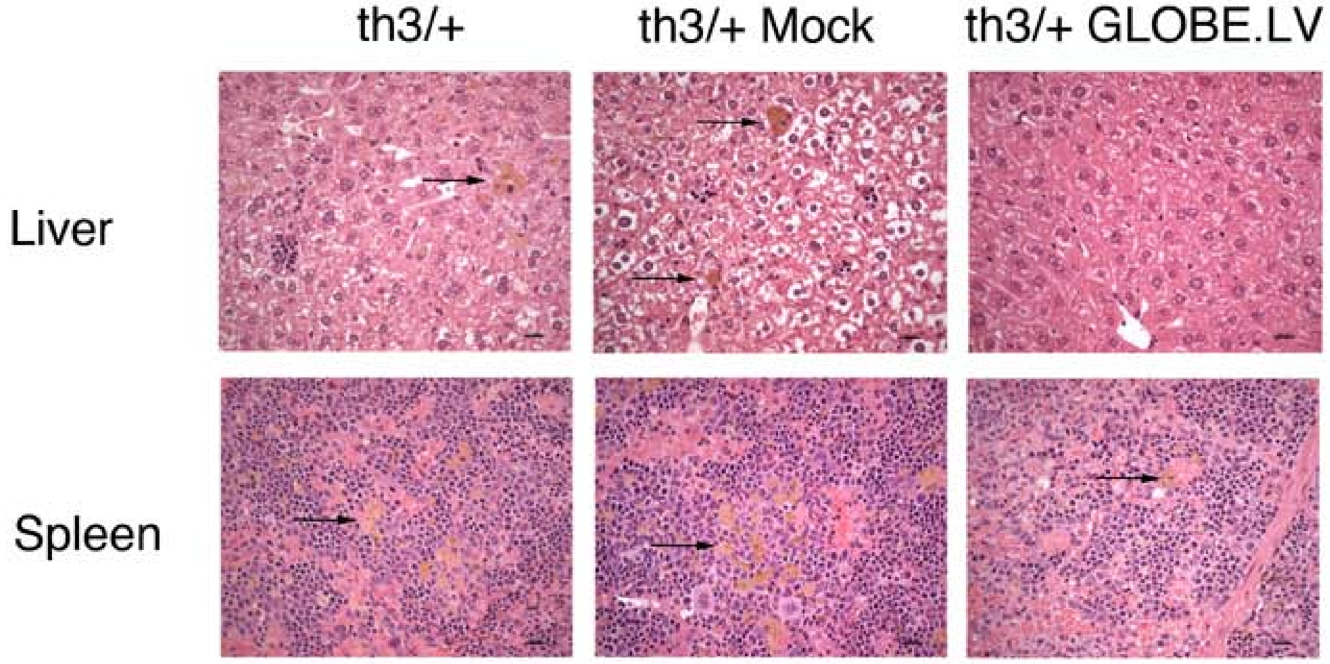

Fig. S2

A

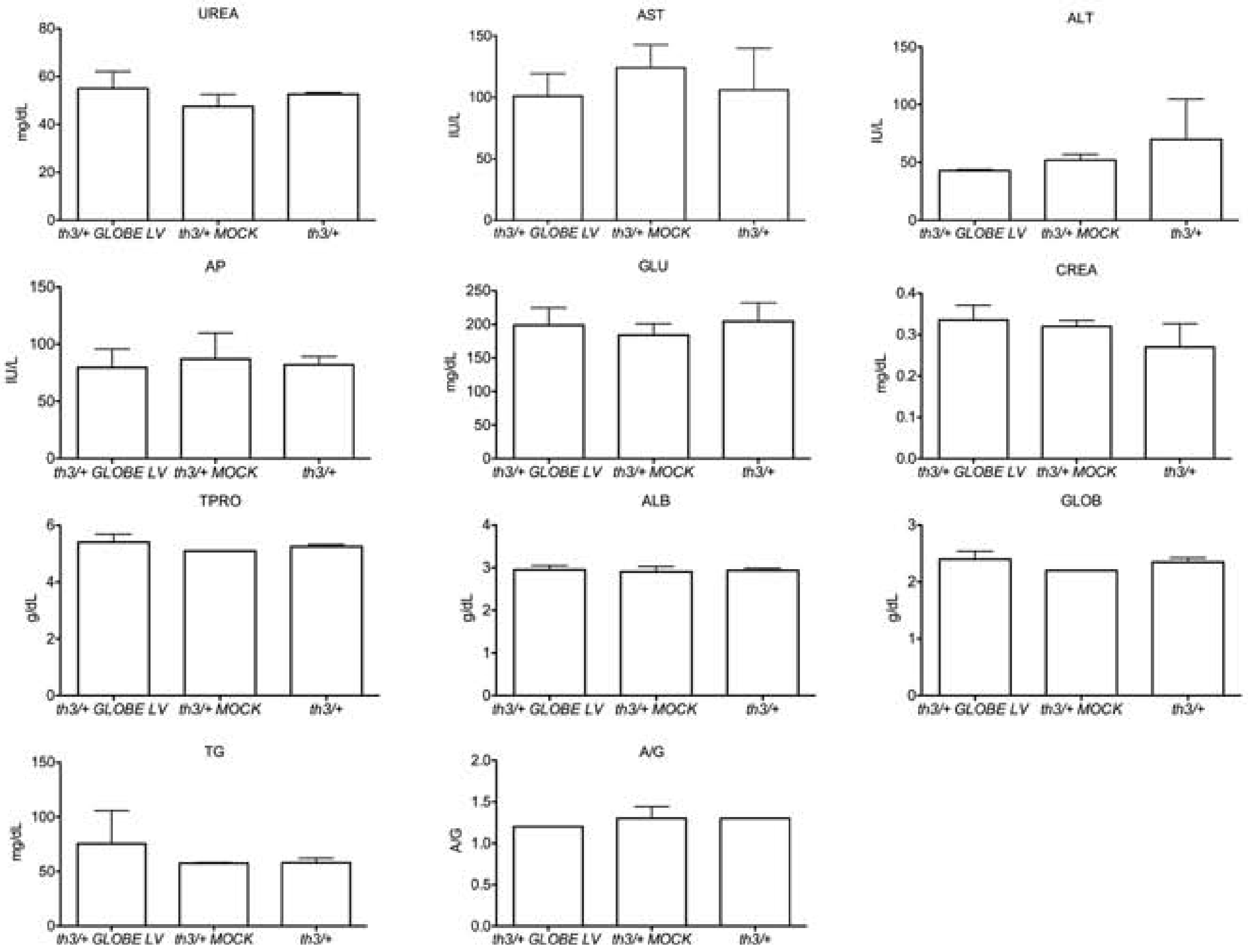
UREA
mg/dL
AST
IU/L
ALT
IU/L
AP
IU/L
GLU
mg/dL
CREA
mg/dL
TPRO
g/dL
ALB
g/dL
GLOB
g/dL
TG
mg/dL
A/G
A/G
th3/+ GLOBE LV
th3/+ MOCK
th3/+

Fig. S3

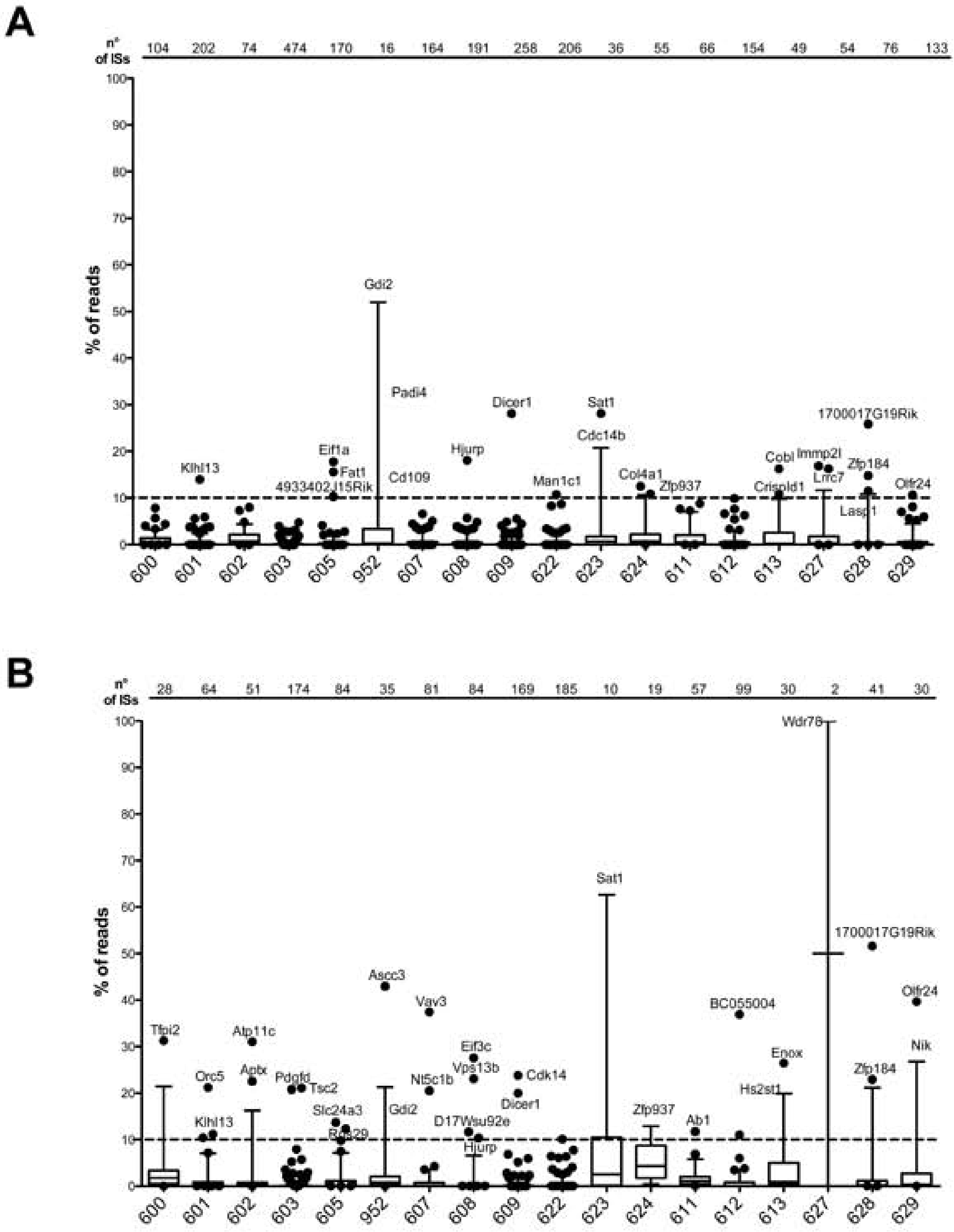
A
B
n° of ISs
% of reads
Gdi2
Padi4
Dicer1
Sat1
Cdc14b
1700017G19Rik
Eif1a
Fat1
4933402J15Rik
Klhl13
Hjurp
Cd109
Man1c1
Col4a1
Zfp937
Cobl
Immp2l
Lrrc7
Zfp184
Crispld1
Lasp1
Olfr24
Wdr78
Asec3
Vav3
Tfpi2
Atp11c
Eif3c
Vps13b
Nt5c1b
Cdk14
Dicer1
Orc5
Aptx
Pdgfd
Tsc2
Slc24a3
D17Wsu92e
BC055004
Enox
Hs2st1
Ab1
Nik

Fig. S4

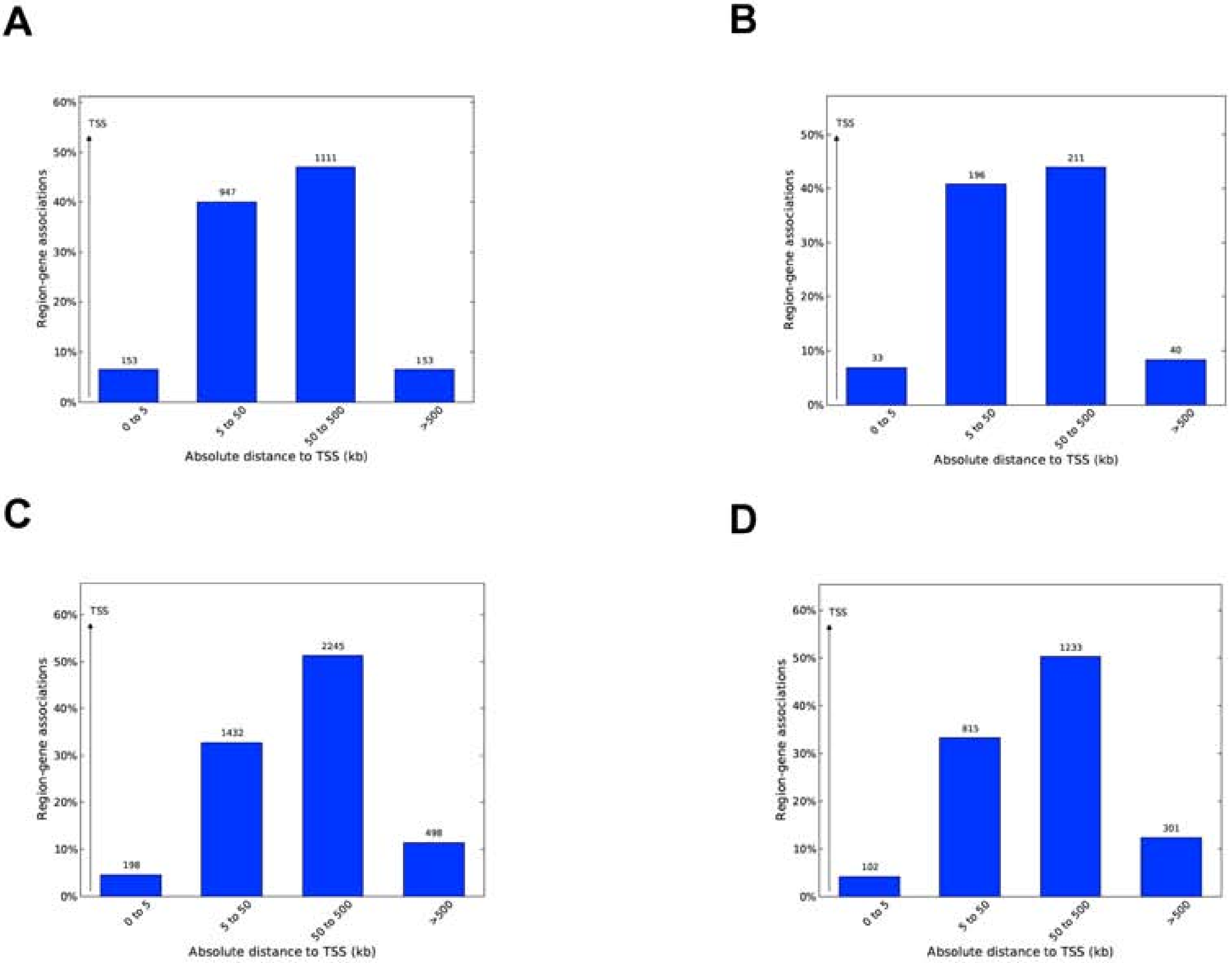
A
B
C
D
TSS
Region-gene associations
Absolute distance to TSS (kb)
0 to 5
5 to 50
50 to 500
>500
0%
10%
20%
30%
40%
50%
60%
153
947
1111
153
33
196
211
40
198
1432
2245
498
102
815
1233
301

Fig. S5

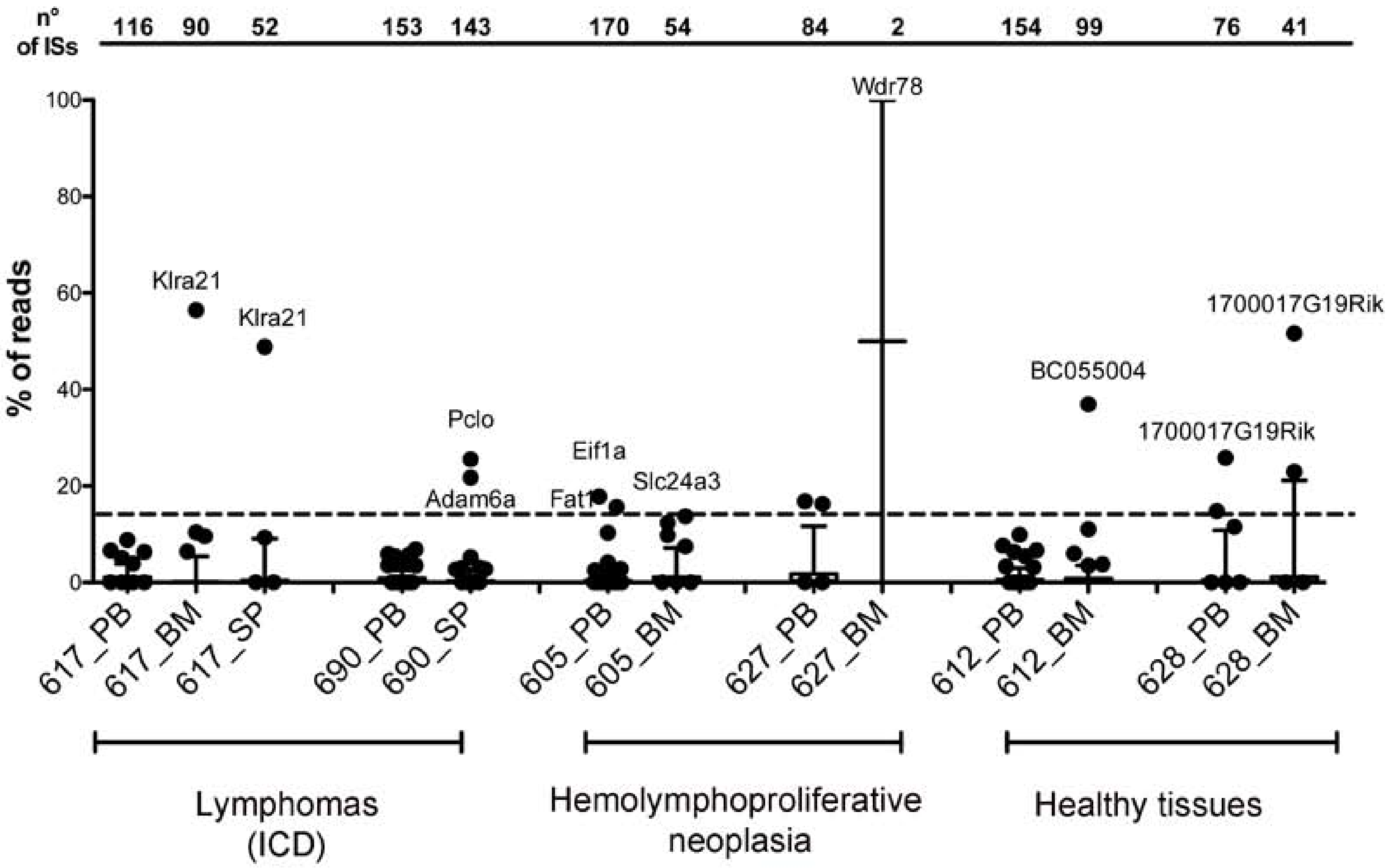
n°
of ISs
116 90 52 153 143 170 54 84 2 154 99 76 41
% of reads
0
20
40
60
80
100
Wdr78
Klra21
Klra21
1700017G19Rik
BC055004
Pclo
1700017G19Rik
Eif1a
Slc24a3
Fat1
Adam6a
617_PB
617_BM
617_SP
690_PB
690_SP
605_PB
605_BM
627_PB
627_BM
612_PB
612_BM
628_PB
628_BM
Lymphomas
(ICD)
Hemolymphoproliferative
neoplasia
Healthy tissues

Fig. S6

A

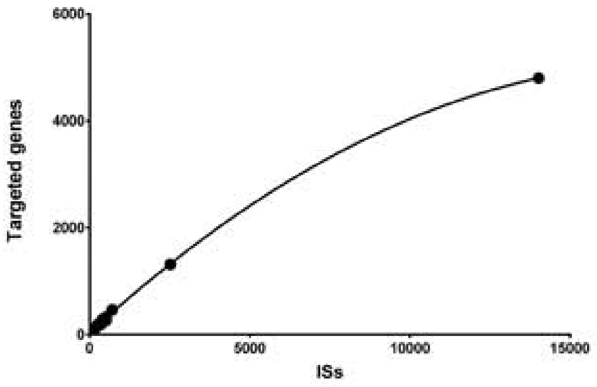

B

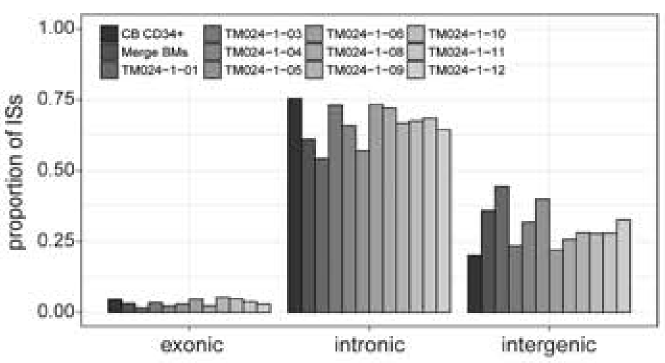

C

Top 100 LV GLOBE-targeted genes *ex-vivo*

PRE-TRANSPLANT
CB CD34⁺ cells

POST-TRANSPLANT
HSCs (from BMs)

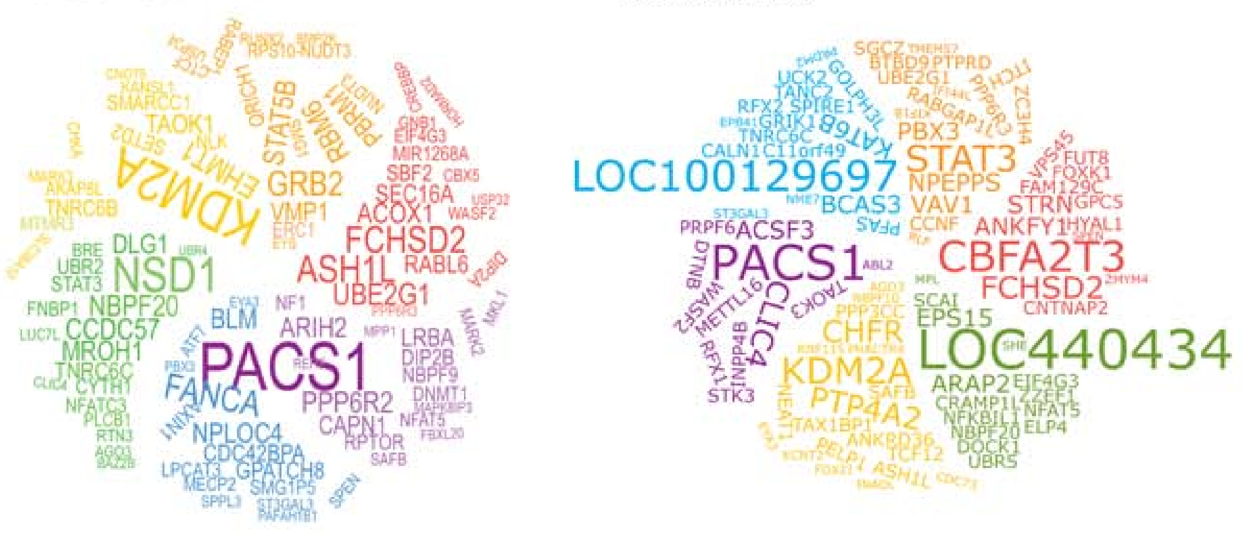

D

Top 20 enriched Gene Ontology categories in LV GLOBE targted-genes in CB CD34+ cells

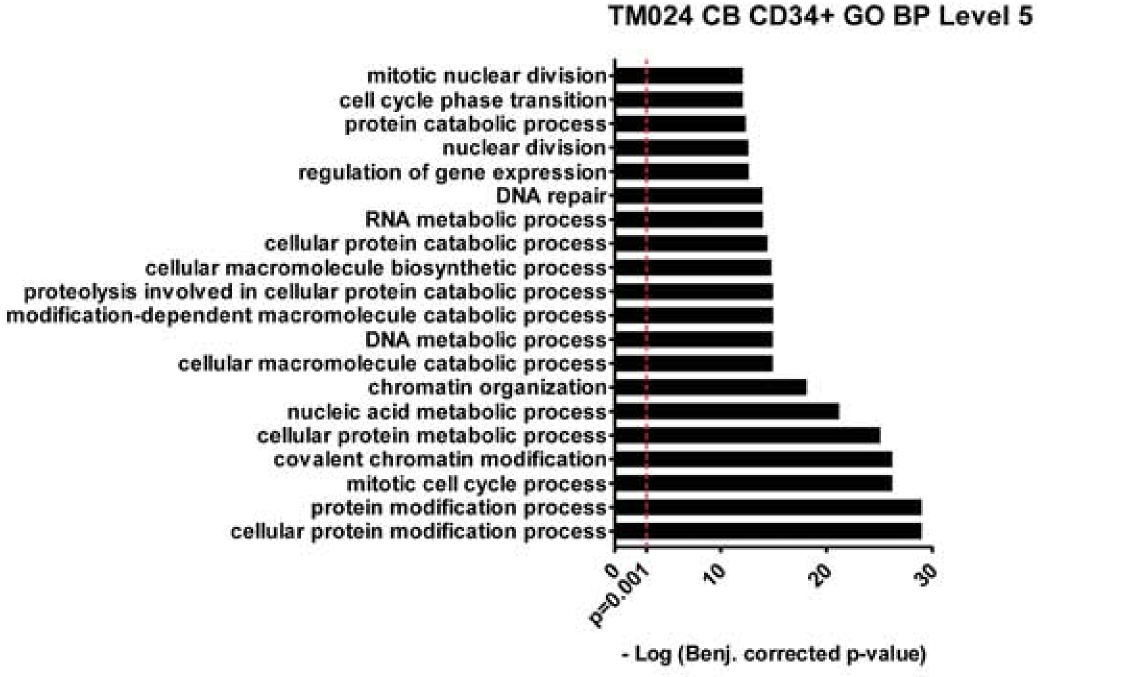

Fig. S7

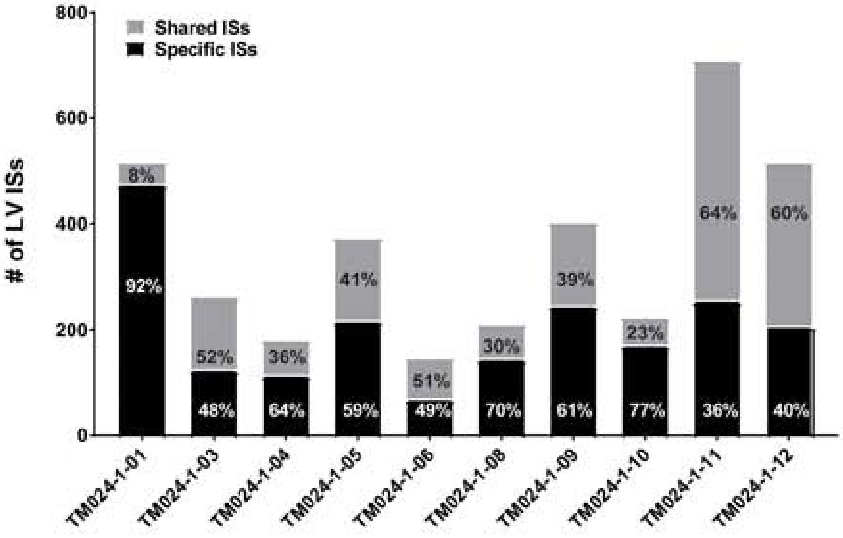
Shared ISs
Specific ISs
# of LV ISs
800
600
400
200
0
8%
92%
52%
48%
36%
64%
41%
59%
51%
49%
30%
70%
39%
61%
23%
77%
64%
36%
60%
40%
TM024-1-01
TM024-1-03
TM024-1-04
TM024-1-05
TM024-1-06
TM024-1-08
TM024-1-09
TM024-1-10
TM024-1-11
TM024-1-12

Fig. S8

Supplemental Figure Legends

Figure S1: Characterization of ß-globin expressing LVs and analysis of transcriptional perturbation

A) VCN of HEL cell clones transduced with GLOBE LV, GATA-GLOBE LV or cHS4-GLOBE LV. Values are shown as mean + SD. Student T-test analysis (two tailed) was applied. **B)** qPCR analysis of ß-globin expression in HEL cell clones transduced with GLOBE LV, GATA-GLOBE LV or cHS4-GLOBE LV induced to erythroid differentiation. Data are expressed relative to GAPDH transcript expression and normalized for VCN. Values are shown as mean + SD. Student T-test analysis (two tailed) was applied. *** = p value < 0.001, **** = p value < 0.0001. **C)** Western Blot analysis of ß-globin expression in HEL cell clones transduced with GLOBE LV, GATA-GLOBE LV or cHS4-GLOBE LV induced to erythroid differentiation. Data are expressed relative to GAPDH protein expression and normalized for VCN. Obtained values were normalized respect to clone GATA-GLOBE 12, loaded on every gel as internal control. Values are shown as mean + SD. T-test analysis (two tailed) was applied. ** = p value < 0.05. **D)** qPCR analysis of genes significantly up- or down-regulated (p value < 0.001) by proviral integration in Uninduced HEL cell clones and Induced HEL cell clones **E**). Red dots indicate the expression value of a gene in the test clone, white dots indicate the expression values of the same gene in control clones.

Figure S2: Extramedullary hematopoiesis in liver and spleen of gene therapy treated mice

Extramedullary hematopoiesis (EMH) in spleen and liver were observed in Th3/+ mice. Related pigmented macrophages (or Kupffer cells) are considered secondary to erythrophagocytosis, Gene therapy treated animals (th3/+ GLOBE.LV) presented signs of efficacy characterized by a prominent reduction of EMH (arrowhead).

Figure S3: Toxicity evaluation of GLOBE LV transduced cells in th3/+ mouse model

A) At termination, PB was collected and used to obtain serum for clinical chemistry. Analyzed parameters were: Urea, Aspartate Aminotransferase (AST), Alanine Aminotransferase (ALT), Alkaline Phosphatase (AP), Glucose (GLU), Creatinine (CREA), (TPRO), Albumin (ALB), Globulin (GLOB), (TG) and the ratio between Albumin and Globulin (A/G). Values are shown as mean + SD. T-test analysis (two tailed) was applied. No difference was observed in all parameters.

Figure S4: Clonal abundance analysis of PB and BM from gene therapy treated mice

Box plot of the percentage of sequence reads (y-axis) for unequivocally mapped IS from peripheral blood at 16 weeks **A)** and bone marrow at termination **B)** is shown. The number of reads for each IS is normalized to the total number of sequence reads from the same time point. IS over the 95 percentiles of dataset are shown as dots distinct from box and whiskers, which are mostly flattened to the bottom of the plot. The total number of IS for each sample is shown on top.

Figure S5: Distribution of LV integrations along the genome of transplanted mice

Integration site (IS) frequency distribution around Transcription Start Site (TSS) of the nearest RefSeq gene, spanning 500 Kb upstream and downstream the TSS**. A)** in vitro cultures **B)** peripheral blood **C)** bone marrow **D)** tumors

Figure S6: Clonal abundance analysis of tumors and controls from gene therapy treated mice

Box plot of the percentage of sequence reads (y-axis) for unequivocally mapped IS from mice which developed lymphomas, hemolymphoproliferative neoplasia and healthy controls. The number of reads for each IS is normalized to the total number of sequence reads from the same time point. IS over the 95 percentiles of dataset are shown as dots distinct from box and whiskers, which are mostly flattened to the bottom of the plot. The total number of IS for each sample is shown on top.

Figure S7. Differential *in vitro* and *ex vivo* integration profile of LV GLOBE.
A) Overall positive correlation between number of ISs and LV-targeted genes in human HSPCs; **B)** Differential distribution of ISs among the intra- and inter-genic regions in pre-transplant CB $CD34^+$ and post-transplant samples; **C)** World cloud graphs of the top 100 LV-targeted genes in HSPCs *in vitro* (left) and *ex vivo* (right). The size of the word is proportional to the number of ISs, while the color has no particular meaning; **D)** The histogram shows the top 20 most significant enriched GO categories in the list of LV-targeted genes in pre-transplant CB $CD34^+$ cells, by DAVID analysis.

Figure S8. ISs shared among different transplanted mice. The histogram shows the portion of ISs specific for each library (black) or common to other libraries (grey).

Table S1: Perturbated genes by different LVs in Uninduced and Induced HEL cells

	Uninduced			Induced		
	GLOBE	GATA-GLOBE	cHS4	GLOBE	GATA-GLOBE	cHS4
Clones	14	11	16	12	10	12
Integrations	36	30	29	35	23	29
Total Genes	92	80	74	87	73	74
p value (of deregulated genes)	0.001	0.001	0.001	0.001	0.001	0.001
Deregulated genes	4	6	1	6	1	6
Up-regulated	3	6	1	6	1	5
Down-regulated	1	0	0	0	0	1
Frequency % (on total genes)	4.35%	7.50%	1.35%	6.90%	1.37%	8.11%

Table S2a : VCN and number of Integration sites in the liquid culture and colony forming units (CFUs)

Sample	Experiment	VCN/cell	Unique IS
CFC	1	9.96	194
LC	1	8.94	267
CFC	2	9.18	70
LC	2	6.36	647
CFC	3	6.35	na
LC	3	6.03	396

Table S2b Percentage of engraftment. VCN and number of integration sites in th3/+ GLOBE LV mice

Mouse	Sex	Engraftment (PB) 16 weeks	VCN/cell PB	Unique IS PB	Engraftment (PB) 12 months	VCN/cell BM	VCN in marked cells (normalized for engraftment)	Unique IS BM	VCN/ cell tumor (SP)	Unique IS tumor (SP)
600	M	72.4	9.63	104	70.2	7.58	10.80	28		
601	M	88.8	3.23	202	90.7	8.19	9.03	64		
602	M	93.3	7.62	74	92.6	6.25	6.75	51		
603	M	86.6	5.41	474	96.5	7.25	7.51	174		
604	M	62.7	4	na	54	2.33	4.31	77		
605	M	78.7	7.44	170	88.1	10.87	12.34	84		
607	M	73.6	6.46	164	55.9	7.97	14.26	81		
608	M	82.1	6.17	191	90	7.95	8.83	84		
609	M	87.2	8.53	258	95.7	10.07	10.52	169		
610	M	40.4	5.23	na	55.6	0.18	0.32	28		
611	M	97.3	8.79	66	99.1	2.25	2.27	57		
612	M	100	4.89	154	99.9	2.35	2.35	99		
613	M	96.8	3.47	49	99	7.67	7.75	30		
614	M	99.9	3.55	na	ICD	ICD	ICD	ICD		
690	M	80.9	15.87	153	ICD	ICD	ICD	ICD	7.82	143
616	F	60.8	4.85	na	60.9	2.8	4.60	47		
617	F	67.3	12.92	116	ICD	16.53	ICD	90	8.69	52
618	F	83.5	7.75	na	80.5	16.39	20.36	na		
619	F	83.4	13.12	na	77.4	5.57	7.20	na		
620	F	49.2	2.51	na	5.4	0.28	5.19	na		
621	F	36.3	1.87	na	53.9	1.67	3.10	na		
622*	F	97.2	1.87	206	98.4	7.68	7.80	185		
623	F	12.4	0.88	36	6.4	0.12	1.88	10		
624	F	91	7.21	55	92.6	14.57	15.73	19		
625	F	97.3	2.54	na	ICD	ICD	ICD	ICD		
626	F	82.6	6.2	na	93.2	0.14	0.15	na		
627	F	15.7	1.03	54	12.8	0.14	1.09	2		
628	F	48.9	1.73	76	71.6	0.62	0.87	41		
629	F	35.4	2.22	133	10.5	0.15	1.43	30		
952	F	22	1.32	16	11.5	0.16	1.39	35		

* sacrified at day 292

Table S2c Percentage of engraftment in th3/+ Mock mice

Mouse	Sex	Engraftment (PB) 16 weeks	Engraftment (PB) 12 months
630	M	64.7	65.5
632	M	81.8	75.5
633	M	70	95
634	M	76.5	89.5
635	M	84.2	95.5
636	M	78.3	87
637	M	88.1	92.2
638	M	100	99.9
639	M	84.2	90.7
640	M	99.9	99.9
641	M	99.9	100
642	M	92.4	93.8
643*	M	86.7	91.7
644	M	90.9	95.6
645	F	96.9	97.8
646	F	95.2	79.2
647	F	0.4	0.2
648	F	0.4	0.6
649	F	0.3	0.04
650	F	0.4	0.01
651	F	5.3	1.8
652	F	1	0.4
653	F	2.4	0.6
654	F	62.2	ICD
655	F	5.4	1.7
656	F	55.9	59.9
657	F	66	52
658	F	5.7	2.8
659	F	22.3	26
692	M	90.9	91.3

* sacrified at day 158

Table S3a: Haematological evaluation th3/+ GLOBE LV group at termination

Anima No.	WBC ($10^3/mm^3$)	RBC ($10^6/mm^3$)	HGB (g/dl)	HCT (%)	MCV ($um^{3)}$	MCH (pg)	MCHC (g/dl)	PLT ($10^3/mm^3$)
600	7.0	8.7	13.0	39.5	45.0	14.9	33.0	1013
601	4.7	8.7	12.8	38.2	44.0	14.7	33.5	1078
602	8.9	8.7	13.3	39.4	45.0	15.2	33.7	1209
603	9.8	9.2	13.1	39.1	43.0	14.3	33.6	1058
604	10.1	8.6	13.1	38.6	45.0	15.3	34.0	1181
605	6.8	9.8	14.0	43.8	45.0	14.3	32.0	858
690	na	na	na	na	na	na	na	na
607	9.3	9.6	14.1	44.3	46.0	14.7	31.8	949
608	8.6	9.3	13.7	42.3	46.0	14.8	32.4	956
609	13.0	8.4	13.0	38.8	46.0	15.5	33.5	2454
610	8.6	9.3	13.9	43.3	46.0	14.8	32.1	896
611	9.3	8.4	12.8	37.7	45.0	15.1	33.8	614
612	6.9	8.7	13.8	40.7	47.0	15.8	33.9	1650
613	9.2	9.2	14.0	41.3	45.0	15.2	34.0	881
614	na	na	na	na	na	na	na	na
952	8.9	8.4	10.2	30.8	37.0	12.1	33.1	2177
616	8.3	8.5	11.1	34.4	41.0	13.0	32.1	1451
617	na	na	na	na	na	na	na	na
618	16.8	10.2	13.7	43.1	42.0	13.4	31.9	1054
619	5.0	7.8	11.3	34.2	44.0	14.6	33.1	842
620	14.6	8.0	9.5	29.4	37.0	11.9	32.2	2004
621	6.2	8.8	11.8	36.2	41.0	13.4	32.6	1280
622	na	na	na	na	na	na	na	na
623	4.2	8.9	13.7	39.7	45.0	15.5	34.6	810
624	6.5	8.6	12.9	38.0	44.0	15.0	33.9	920
625	na	na	na	na	na	na	na	na
626	7.2	8.5	12.8	37.8	44.0	15.0	33.8	920
627	2.7	7.1	12.9	36.3	51.0	18.1	35.5	911
628	8.1	8.8	13.7	38.0	43.0	15.6	35.9	968
629	3.0	8.6	14.2	39.4	46.0	16.6	36.0	1040

Table S3b: Haematological evaluation th3/+ Mock group at termination

Anima No.	WBC ($10^3/mm^3$)	RBC ($10^6/mm^3$)	HGB (g/dl)	HCT (%)	MCV ($um^{3)}$	MCH (pg)	MCHC (g/dl)	PLT ($10^3/mm^3$)
630	6.8	3.1	4.3	12.1	39.0	13.8	35.7	1054
692	14.2	7.0	9.1	27.3	39.0	13.0	33.3	2212
632	13.6	6.5	8.5	25.5	39.0	13.0	33.2	1862
633	15.5	7.3	9.5	28.9	40.0	13.0	32.9	1990
634	5.4	2.4	3.6	9.9	41.0	14.8	36.2	773
635	23.2	6.3	8.4	25.3	40.0	13.3	33.1	1985
636	31.0	7.3	9.9	31.3	43.0	13.6	31.7	1892
637	34.6	6.9	10.0	29.2	42.0	14.5	34.4	2256
638	35.0	6.6	9.7	29.5	45.0	14.7	33.0	2235
639	27.1	6.5	9.4	27.5	42.0	14.4	34.2	2129
640	21.5	7.0	10.1	29.8	43.0	14.4	33.9	1902
641	16.1	6.6	9.5	27.7	42.0	14.4	34.4	2090
642	17.7	6.9	9.8	29.0	42.0	14.2	33.9	2100
643	na	na	na	na	na	na	na	na
644	16.3	6.6	9.4	27.8	42.0	14.3	33.8	1800
645	14.0	7.3	9.9	28.9	39.0	13.5	34.1	2286
646	21.2	7.7	10.0	30.0	39.0	13.1	33.4	2133
647	16.3	7.0	9.5	28.4	41.0	13.6	33.4	1935
648	17.7	7.2	9.7	29.3	41.0	13.5	33.1	2006
649	37.6	7.0	10.1	30.9	44.0	14.4	32.7	2210
650	35.1	6.8	9.3	28.5	42.0	13.7	32.5	2231
651	15.0	6.8	9.6	27.3	40.0	14.2	35.3	1915
652	14.7	6.8	9.6	27.2	40.0	14.2	35.3	1717
653	15.8	6.3	9.0	26.2	42.0	14.4	34.4	1759
654	na	na	na	na	na	na	na	na
655	14.0	6.9	9.5	27.6	40.0	13.9	34.5	1977
656	28.1	6.7	9.9	27.5	41.0	14.7	35.9	2032
657	0.0	6.9	9.7	27.9	41.0	14.1	34.7	2054
658	0.0	7.0	9.8	28.5	41.0	14.1	34.5	2123
659	0.0	7.0	10.1	28.9	41.0	14.4	34.9	2073

Table S3c: Haematological evaluation th3/+ group at termination

Anima No.	WBC ($10^3/mm^3$)	RBC ($10^6/mm^3$)	HGB (g/dl)	HCT (%)	MCV ($um^{3)}$	MCH (pg)	MCHC (g/dl)	PLT ($10^3/mm^3$)
660	4.2	6.2	5.4	24.2	39.0	8.7	22.4	1347
661	25.1	8.0	10.3	31.4	39.0	12.9	32.9	2467
662	18.2	7.2	9.4	27.9	39.0	13.1	33.6	2128
663	14.3	7.1	9.3	28.0	40.0	13.2	33.2	2078
664	18.1	5.7	8.3	24.6	43.0	14.5	33.7	1933
665	18.2	5.9	8.5	25.5	44.0	14.5	33.4	1943
666	19.6	6.2	8.9	26.7	43.0	14.4	33.2	1971
935	29.4	6.9	9.8	30.1	44.0	14.2	32.5	1655
691	38.1	6.9	10.0	30.7	44.0	14.5	32.7	2078
669	36.7	6.8	9.6	28.7	42.0	14.1	33.5	2302
670	18.9	6.7	9.5	28.1	42.0	14.3	33.8	1854
671	16.9	6.5	9.4	17.6	42.0	14.5	34.2	1821
672	15.7	6.6	9.6	27.5	42.0	14.5	34.9	2096
673	17.9	6.8	9.8	28.6	42.0	14.4	34.3	2108
674	15.0	7.3	10.1	29.2	40.0	13.9	34.6	1957
675	16.2	7.0	9.2	27.2	39.0	13.2	33.9	2220
676	16.9	7.1	9.4	27.6	39.0	13.3	34.0	2224
677	29.9	7.1	9.7	30.0	42.0	13.7	32.2	1790
678	24.7	7.5	10.3	31.1	42.0	13.9	33.3	2385
679	25.3	7.5	10.4	31.2	42.0	14.0	33.5	2335
680	29.6	7.6	10.0	30.7	41.0	13.2	32.5	2347
681	13.8	6.8	9.5	27.5	40.0	13.9	34.5	1899
682	13.2	6.5	9.0	25.9	40.0	13.9	34.6	1705
683	16.0	6.3	9.2	26.4	42.0	14.5	34.7	1726
684	21.7	7.1	10.1	30.0	42.0	14.2	33.8	1523
685	25.1	7.1	10.6	29.6	42.0	14.8	35.6	2105
686	28.5	7.1	10.0	28.8	41.0	14.1	34.6	2147
687	27.6	6.1	8.8	27.0	44.0	14.4	32.4	1609
688	28.4	6.8	9.6	28.6	42.0	14.2	33.6	2290
689	36.4	6.6	9.9	29.5	45.0	15.0	33.5	2209

Table S4: RTCGD analysis

Sample	in vitro	PB	BM	SP
n° of genes tested	1290	1765	1010	175
n° of genes in RTCGD	286	401	209	30
% of genes in RTCGD	22.2	22.7	20.7	17.1

Table S5. ISs distribution along the human genome

Sample	Raw reads	Mapped reads	# LV ISs	Inter-genic	Intra-genic (Exons)	Intra-genic (Introns)	# LV-targeted genes
**CB CD34$^+$**	4.611.257	3.287.603	14.023	0.05	0.2	0.75	4.802
**TM024-1-01**	2.384.753	1.056.020	515	0.02	0.44	0.54	265
**TM024-1-03**	693.561	438.794	263	0.03	0.24	0.73	191
**TM024-1-04**	556.507	402.491	180	0.02	0.32	0.66	120
**TM024-1-05**	879.491	598.145	372	0.03	0.4	0.57	201
**TM024-1-06**	809.812	525.727	146	0.05	0.22	0.73	107
**TM024-1-08**	876.857	583.019	210	0.02	0.26	0.72	151
**TM024-1-09**	950.808	757.092	402	0.05	0.28	0.67	287
**TM024-1-10**	674.862	277.620	223	0.05	0.28	0.68	149
**TM024-1-11**	1.222.899	727.632	709	0.04	0.28	0.68	465
**TM024-1-12**	677.594	475.264	515	0.03	0.33	0.64	327
**TM024 merge BMs**	9.727.144	5.844.941	2.518	0.03	0.35	0.62	1.316

Table S6. ISs and targeted genes in common between pre- and post-transplant samples.

Targeted genes	TM024 1-01	TM024 1-03	TM024 1-04	TM024 1-05	TM024 1-06	TM024 1-08	TM024 1-09	TM024 1-10	TM024 1-11	TM024 1-12
Tot	265	191	120	201	107	151	287	149	465	327
Specific	119	48	35	52	27	45	90	47	124	91
**Shared with CB_CD34+**	146	143	85	149	80	106	197	102	341	236
**Shared with CB_CD34+ (%)**	55%	75%	71%	74%	75%	70%	69%	68%	73%	72%

Individual ISs	TM024 1-01	TM024 1-03	TM024 1-04	TM024 1-05	TM024 1-06	TM024 1-08	TM024 1-09	TM024 1-10	TM024 1-11	TM024 1-12
Tot	515	263	180	372	146	210	402	223	709	515
Specific	515	262	180	371	146	210	400	223	708	515
**Shared with CB_CD34+**	0	1	0	1	0	0	2	0	1	0
**Shared with CB_CD34+ (%)**	0.0%	0.4%	0.0%	0.3%	0.0%	0.0%	0.5%	0.0%	0.1%	0.0%

Table S7. Shared ISs between individual pre- and post-transplant samples

	**TM024 1-01**	**TM024 1-03**	**TM024 1-04**	**TM024 1-05**	**TM024 1-06**	**TM024 1-08**	**TM024 1-09**	**TM024 1-10**	**TM024- 1-11**	**TM024 1-12**	**CB CD34$^+$**
**TM024-1-01**	472	0	1	4	0	11	24	3	23	12	-
**TM024-1-03**	0	124	13	14	6	14	47	12	129	77	1
**TM024-1-04**	1	13	114	17	3	19	35	13	62	44	-
**TM024-1-05**	4	14	17	213	8	5	44	5	147	119	1
**TM024-1-06**	0	6	3	8	66	28	49	17	77	54	-
**TM024-1-08**	11	14	19	5	28	145	48	26	55	49	-
**TM024-1-09**	24	47	35	44	49	48	242	30	137	124	2
**TM024-1-10**	3	12	13	5	17	26	30	171	52	32	-
**TM024-1-11**	23	129	62	147	77	55	137	52	251	287	1
**TM024-1-12**	12	77	44	119	54	49	124	32	287	206	-
**CB CD34$^+$**	0	1	0	1	0	0	2	0	1	0	14.023
